# Threading light through dynamic complex media

**DOI:** 10.1038/s41566-025-01642-z

**Published:** 2025-03-14

**Authors:** Chaitanya K. Mididoddi, Robert J. Kilpatrick, Christina Sharp, Philipp del Hougne, Simon A. R. Horsley, David B. Phillips

**Affiliations:** 1 Physics and Astronomy, University of Exeter, Exeter, UK; 2 Université de Rennes, CNRS, IETR - UMR 6164, Rennes, France

**Keywords:** Imaging and sensing, Adaptive optics, Imaging techniques

## Abstract

The dynamic scattering of light impacts sensing and communication technologies throughout the electromagnetic spectrum. Here we introduce a new way to control the propagation of light through time-varying complex media. Our strategy is based on the observation that in many dynamic scattering systems, some parts of the medium will change configuration more slowly than others. We experimentally demonstrate a suite of new techniques to identify and guide light through the more temporally stable channels within dynamic scattering media—threading optical fields around multiple highly dynamic pockets hidden at unknown locations inside. We first show how the temporal fluctuations in scattered light can be suppressed by optimizing the wavefront of the incident field. Next, we demonstrate how to accelerate this procedure by two orders of magnitude using a physically realized form of adjoint gradient descent optimization. Finally, we show how the time-averaged transmission matrix reveals a basis of temporal fluctuation eigenchannels that can be used to increase the stability of beam shaping through time-varying complex media such as bending multimode fibres. Our work has potential future applications to a variety of technologies reliant on general wave phenomena subject to dynamic conditions, from optics to microwaves and acoustics.

## Abstract (web summary)

Guiding light around dynamic regions of a scattering object by means of propagating light through the most ‘stable’ channel within a moving scattering medium is demonstrated, potentially advancing fields such as deep imaging in living biological tissue and optical communications through turbulent air and underwater.

## Main

Optical scattering randomly redirects the flow of light. It is a ubiquitous phenomenon that has wide-ranging effects. Since imaging relies on light travelling in straight lines from a scene to a camera, scattering prevents image formation through fog and precludes high-resolution microscopy inside biological tissue^1,2^. Scattering also impairs optical communications through air and water, and disrupts the transmission of microwave and radio signals^3^. Overcoming the adverse effects of light scattering is an extremely challenging problem^4^. Nonetheless, owing to its prominence, substantial progress has been made over the past decades^5^.

When light propagates through a strongly scattering environment (also known as a ‘complex’ medium^1^), the wavefront of the incident optical field is distorted, corrupting the spatial information that it carries. Elastic scattering from a static medium is deterministic, meaning that the precise way in which light has been perturbed can be characterized and subsequently corrected. By sending a series of probe measurements through the medium, a digital model of its effect on light can be created: represented by a linear matrix operator known as a transmission matrix (TM)^6^. The TM reveals how to pre-distort an input optical field so that it evolves into a user-defined state at the output—a technique known as wavefront shaping^7^.

Despite these successes, control of light through time-varying complex media remains a largely open problem^2^. Evidently, wavefront shaping can only be successfully applied if the medium in question stays predominantly stationary for the time taken to make probe measurements and apply a wavefront correction. Yet many application scenarios feature complex media that rapidly fluctuate on a timescale of milliseconds or faster^8^—rendering wavefront shaping approaches exceedingly difficult. So far, the main strategies to control light through moving complex media have focused on achieving the task of wavefront shaping as quickly as possible^8–16^. Approaches rely on ultra-fast beam shaping^17–23^ or reducing the number of probe measurements by, for example, spectral multiplexing^21,24^ or exploiting prior knowledge about the scattering medium^25–29^.

Here we introduce an alternative way to control the propagation of light through dynamic scattering media. We begin by classifying complex media into three categories, based on the type of motion exhibited over the timescale required for wavefront shaping, *τ*_ws_. Class 1 represents static complex media that remain fixed over time *τ*_ws_—established TM-based methods can be applied in this case. Class 2 represents moving complex media, which undergo substantial motion everywhere over time *τ*_ws_ and elude current wavefront shaping approaches. There is a third class of medium that falls between classes 1 and 2. Class 3 comprises partially moving scattering media, which, over the timescale *τ*_ws_, exhibit localized time-varying pockets embedded within a comparatively static medium. This situation describes, for example, slowly moving tissue through which the small capillaries conducting blood flow typify faster changing regions, and pockets of turbulent air above hot chimneys within calmer air over a city skyline. In this article, we focus on how to identify light fields that thread through networks of static material within such partially moving complex media.

## Results

When a light field **u** is incident on a dynamic medium, the time-dependent transmitted field **v**(*t*) is given by

1 $${\bf{v}}(t)={\bf{T}}(t){\bf{u}},$$

where **T**(*t*) is the time-dependent TM of the medium, and **u** and **v** are complex column vectors. Our aim is to find an input **u** that minimizes the temporal fluctuations in the output field **v**(*t*) by analysing externally scattered light.

**Fig. 1:**
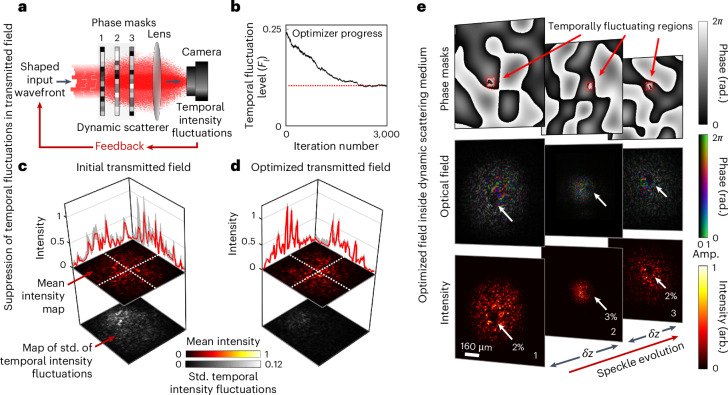
Unguided optimization. **a**, Schematic of experimental set-up. An input wavefront is iteratively modified to reduce the output temporal intensity fluctuations. **b**, A plot of temporal fluctuation level (*F*_*l*_) as a function of iteration number throughout the optimization procedure. Convergence plateaus (red dashed line) after ~2,500 iterations. **c**, Output intensity fluctuations for a randomly chosen input field. Upper heat maps show the mean intensity of transmitted light, and lower heat maps show the standard deviation of the intensity fluctuations. The line plots show line profiles through the output field along the lines marked with white hatched lines, with mean intensity (red line) and fluctuations about the mean (grey shading). Std., standard deviation. **d**, Equivalent plot to **c** but showing the suppressed output intensity fluctuations for an optimized input field. **e**, Measured shape of the optimized field inside the dynamic scattering sample. Top row: examples of the three phase planes forming the artificial scattering system, with a small fluctuating patch on each plane highlighted by a red box. Middle row: optimized optical field incident on each plane. Bottom row: corresponding intensity incident on each plane (max intensity on each plane is normalized to 1). We observe a low-intensity region on the three dynamic patches, highlighted by white arrows (percentage of intensity passing through each patch is quoted). The distance between the planes is *δz* = 5 cm. amp., amplitude; rad., radians; arb., arbitrary unit.

To begin experimentally investigating this scenario, we emulate a three-dimensional time-varying forward scattering medium using a cascade of three computer-controlled diffractive optical elements, each separated by free space, as shown in Fig. 1. Cascades of phase planes can emulate atmospheric turbulence^30,31^ and mimic multiple scattering samples^32–35^. In practice, our set-up is implemented using multiple reflections from a liquid crystal spatial light modulator (SLM), allowing the phase profiles to be arbitrarily digitally reconfigured. We choose this test bed as it is a versatile way to control the number and location of dynamic regions for proof-of-principle experiments.

As shown in Fig. 1e (top row), we show a static random phase pattern on each phase screen, spatially distorting optical signals flowing through the system. On each plane, we also define an area within which the phase profile is programmed to randomly fluctuate in time. A second SLM is used to shape the incident light, and a digital camera records the level of temporal intensity fluctuations in transmitted light. We quantify these fluctuations by calculating the temporal fluctuation level ${F}_{l}={\bar{\sigma }}_{I}/{\bar{\mu }}_{I}$, where ${\bar{\sigma }}_{I}$ denotes the standard deviation of the time-fluctuating intensity, averaged over all illuminated camera pixels, and ${\bar{\mu }}_{I}$ is the average transmitted intensity. For example, *F*_*l*_ = 0 for an unchanging transmitted field, and *F*_*l*_ = 1 for a series of uncorrelated Rayleigh speckle patterns (Methods). Here we use the term ‘fluctuations’ to refer to temporal fluctuations.

### Unguided optimization

We first pursue a straightforward optimization method: iterative adjustment of the input field **u** to suppress the level of temporal intensity fluctuations (*F*_*l*_) in the transmitted field. Our approach is shown schematically in Fig. 1a (Methods and Supplementary Section 1). Figure 1b shows a typical optimization convergence curve, which plateaus at *F*_*l*_ ~0.1. Noiseless simulations of this experiment reproduce a similar convergence plateau (Supplementary Section 8). We speculate that this may be because we optimize only the phase of the input field in this experiment. Figure 1c,d shows examples of the output temporal fluctuations of a randomly chosen initial trial field (*F*_*l*_ = 0.25) and an optimized field (*F*_*l*_ = 0.1), respectively. See also Supplementary Video 1, which shows that intensity fluctuations have been heavily suppressed.

The artificial nature of our dynamic scattering medium renders it possible to directly observe the evolution of the optimized field inside by digitally ‘peeling back’ the outer scattering layers (Supplementary Section 2). We find the optimized field takes the form of a speckle pattern that evolves to exhibit near-zero intensity on fluctuating regions on each plane (Fig. 1e, bottom row)—thus almost entirely avoiding these dynamic areas.

Our unguided optimization strategy is analogous to the first methods used to shape light through static scattering media^7^, and as such may be improved using more advanced algorithms^36,37^. Furthermore, the form of the objective function can be arbitrarily chosen. For example, intensity shaping terms could potentially be included to simultaneously reduce fluctuations and shape the output^38,39^. However, undirected optimization is a relatively slow process requiring many iterations to converge (~2,500 in our experiment). Therefore, we next ask: is there a way to find optimized fields more rapidly?

### Physical adjoint optimization

In our first strategy, on each iteration, we measure how one single spatial component of the input field should be adjusted to reduce *F*_*l*_. We now describe how to simultaneously measure how all spatial components should be adjusted in parallel. Our approach can be understood as gradient descent optimization using a physical realization of fast adjoint methods—which enable the efficient determination of the gradient of an objective function with respect to the optimization variables.

**Fig. 2:**
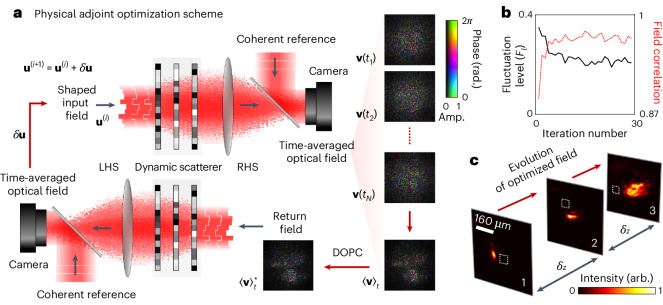
Physical adjoint optimization. **a**, Schematic of experimental set-up. On iteration *i*, an input field **u**^(*i*)^ is transmitted through the dynamic medium from the LHS. The output field is coherently time-averaged on the RHS—the schematic shows output fields recorded at individual times: **v**(*t*_1_), **v**(*t*_2_) ⋯ **v**(*t*_*N*_) (where *N* is the total number of recorded output fields). These measurements are averaged to yield ${ \langle {\bf{v}} \rangle }_{t}$. DOPC is carried out to transmit the phase conjugate of ${ \langle {\bf{v}} \rangle }_{t}$ back through the medium. The resulting field emerging on the LHS is then coherently time-averaged, and used to calculate *δ***u**, such that the input of the next iteration (*i* + 1) is given by **u**^(*i*+1)^ = **u**^(*i*)^ + *δ***u**. **b**, A plot of temporal fluctuation level (*F*_*l*_) (black line) as a function of iteration number throughout the optimization procedure. In this scheme, convergence is reached after ~15 iterations. We also plot the normalized field correlation between all output fields at each iteration (dashed red line). **c**, The experimentally recorded intensity of the optimized field arriving at the three phase planes (max intensity at each plane is normalized to 1). The white squares indicate the location of the moving region on each plane. We see that the optimized field avoids these moving regions of the sample.

Our scheme is shown in Fig. 2a (Methods and Supplementary Section 4). To render an adjoint optimization approach feasible, it is necessary to modify the optimization objective function and send light back and forth in both directions through the dynamic scattering medium (reminiscent of work placing a scattering medium inside a laser cavity^9^). We now aim to maximize the correlation *C* between all *N* measured output fields over time, given by

2 $$C=\frac{1}{N}{\left\vert \mathop{\sum }\limits_{t = 1}^{T}\mathop{\sum }\limits_{t{\prime} = 1}^{T}\left[{{\bf{v}}}^{\dagger }(t)\cdot {\bf{v}}(t{\prime} )\right]\right\vert }^{2}.$$

See Supplementary Section 3 for derivation of this method and proof of its convergence.

Figure 2b shows the increase in the objective function throughout the optimization process. For comparison with our first strategy, we also monitor the level of temporal fluctuations *F*_*l*_. After only ~15 iterations the procedure converges. See also Supplementary Video 2. Here we attribute the higher level of residual fluctuations to additional noise induced by the asynchronous uncontrolled flickering of the three SLMs used in this experiment. Supplementary Section 8 provides supporting simulations and discussion of the noise floor. Once again looking inside the dynamic medium, we see that we have found a more smoothly varying optical field that avoids the moving regions, as shown in Fig. 2c—an effect also reproduced in simulations (Supplementary Section 8).

This adjoint optimization strategy is reminiscent of iterative time reversal^40^ and recently proposed in situ methods to train photonic neural networks^41^. Indeed, our work may be considered one of the first real-world implementations of a photonic adjoint optimization routine—a challenging yet powerful method to realize experimentally^42^.

### Temporal fluctuation eigenchannels of the time-averaged TM

**Fig. 3:**
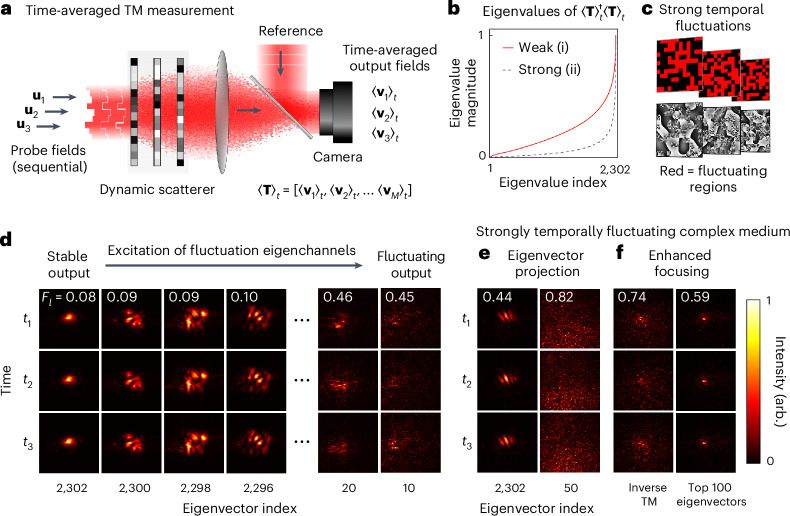
Time-averaged TM. **a**, Schematic of experimental set-up. A sequence of orthogonal input probe fields are individually transmitted through the medium, for example, **u**_1_, **u**_2_, **u**_3_. For each input, the corresponding time-averaged output field is recorded, for example, ${ \langle {{\bf{v}}}_{1} \rangle }_{t}$, ${ \langle {{\bf{v}}}_{2} \rangle }_{t}$, ${ \langle {{\bf{v}}}_{3} \rangle }_{t}$, and arranged column-wise to build the time-averaged TM 〈**T**〉_*t*_. **b**, The magnitudes of the eigenvalues of ${\langle {\bf{T}}\rangle }_{t}^{\dagger }{\langle {\bf{T}}\rangle }_{t}$ for a weakly (i) and strongly (ii) fluctuating dynamic medium. Both are arranged in ascending order and normalized to a maximum value of 1. The weakly fluctuating medium is the same as used in the earlier experiments. **c**, An example of the strongly fluctuating medium, with moving regions highlighted in red. **d**, Excitation of selected temporal fluctuation eigenchannels in the weakly fluctuating medium. Each column shows the output when the medium is illuminated with different eigenvectors. Each row shows the output at a different time—that is, for three different configurations of the dynamic regions of the medium. We see that the high-index eigenvectors are stable with respect to these movements, while the low-index eigenvectors are not. **e**, Eigenvector projection through a strongly fluctuating medium. **f**, Enhanced focusing through strongly fluctuating scattering media. Left column: focusing using the conventional inverse TM, which is measured while the medium fluctuates. We see a poor contrast focus, which fluctuates strongly as the medium reconfigures. Right column: focusing using a linear combination of the top 100 most stable eigenvectors of ${\langle {\bf{T}}\rangle }_{t}^{\dagger }{\langle {\bf{T}}\rangle }_{t}$. Here we see that the contrast and stability of the output focus are improved. The peak intensity is increased by a factor of 2.8 when using the top 100 eigenvectors. In **d**–**f**, each column is separately normalized to the peak intensity across the three panels.

So far, we have focused on strategies to find a single stable channel. We now consider how to determine a set of stable channels that all navigate around dynamic regions inside the medium. Our approach makes use of the information stored in the time-averaged TM of a fluctuating optical system, 〈**T**〉_*t*_. Figure 3a shows how 〈**T**〉_*t*_ is measured (Methods and Supplementary Section 5).

〈**T**〉_*t*_ reveals the input fields that deliver high levels of coherently time-averaged energy to the output. Finding such input fields can be represented as an eigenvalue problem by noting that the total intensity of the time-averaged field arriving at the output, 〈*P*〉_*t*_, can be expressed as

3 $${\langle P\rangle }_{t}={\langle {\bf{v}}\rangle }_{t}^{\dagger }{\langle {\bf{v}}\rangle }_{t}={{\bf{u}}}^{\dagger }{\langle {\bf{T}}\rangle }_{t}^{\dagger }{\langle {\bf{T}}\rangle }_{t}{\bf{u}}.$$

Therefore, the eigenvectors of matrix ${\langle {\bf{T}}\rangle }_{t}^{\dagger }{\langle {\bf{T}}\rangle }_{t}$ with the largest eigenvalues represent input fields that deliver the highest intensity of the time-averaged output fields. These eigenvectors also correspond to input fields that interact least with the dynamic regions inside the medium, under the assumption that the internal fluctuations of the medium are large enough to randomize the phase of dynamically scattered light, meaning that fluctuating fields can be effectively ‘time-averaged away’ (Supplementary Section 12). We term this basis of eigenvectors the ‘temporal fluctuation eigenchannels’ of the dynamic medium. This concept links to our previous approach: we show in Supplementary Section 3 that when using the objective function given in equation (2), physical adjoint optimization finds the most stable temporal fluctuation eigenchannel of the time-averaged TM.

Figure 3b shows the distribution of absolute eigenvalues of ${\langle {\bf{T}}\rangle }_{t}^{\dagger }{\langle {\bf{T}}\rangle }_{t}$, arranged in ascending order. We compare two 3-plane dynamic samples with different numbers of moving regions: (i) has a single dynamic patch on each plane similar to before; (ii) has randomly placed fluctuating patches covering approximately half of the area of each plane. We show in Supplementary Section 11 that medium (i) has at least one stable temporal fluctuation eigenchannel that avoids all dynamic patches, while medium (ii) has no completely stable channels.

We first demonstrate excitation of the fluctuation eigenchannels through the more weakly fluctuating sample, shown in Fig. 3d. We see that the transmitted fields corresponding to high eigenvalues remain stable. Conversely, the transmitted fields corresponding to low eigenvalues vary with time—as these modes interact strongly with the moving parts of the medium. See also Supplementary Video 3.

We now turn our attention to the more strongly fluctuating medium. Figure 3e shows the stability of transmitted fields when exciting channels with high and low eigenvalues. As expected, even light propagating through the most stable eigenchannel exhibits non-negligible output fluctuations over time, indicating that we have not found any fields that thread perfectly around all dynamic regions. Despite this, we find that a marked improvement in focusing at the output is possible using the information stored in the time-averaged TM. Figure 3f shows a focus created using conventional wavefront shaping, where the medium freely fluctuates throughout TM measurement (left column), compared with focusing using a sub-basis formed from the top 100 most stable temporal fluctuation eigenchannels (right column)—see Supplementary Section 6 and Supplementary Video 4 for details. Both the contrast and stability of the focus are enhanced. Beyond focusing, more elaborate beam shaping may also be possible using this stable sub-basis^43^.

The time-averaged TM is related to several previously introduced matrix operators connected to physical quantities in scattering media^6,44–47^. In particular, it has similar properties to the TM of a static medium with inhomogeneous absorption^39,48,49^. As fluctuations in the medium go to zero, the time-averaged TM tends to the conventional TM, and the temporal fluctuation eigenchannels tend to the transmission eigenchannels of a static scattering medium^50,51^. The ‘deposition matrix’^47^ and the ‘generalized Wigner-Smith operator’^44,52^ are both also capable of revealing light fields that circumnavigate predetermined regions within a complex medium. However, only the time-averaged TM does so without requiring access to internal fields within the medium^47^ or the measurement of an entire TM while the medium is held static^44^.

### Stable light transmission through flexing optical fibre

Up to this point, we have considered samples with well-defined moving regions embedded within static complex media. We now explore a more general scenario in which an entire medium deforms, leaving no clearly identifiable locations that remain static. To investigate this case, we search for stable channels within a step-index multimode fibre (MMF) as it is gradually bent—here all transmitted spatial modes will interact with the core-cladding boundary of the fibre as it flexes.

**Fig. 4:**
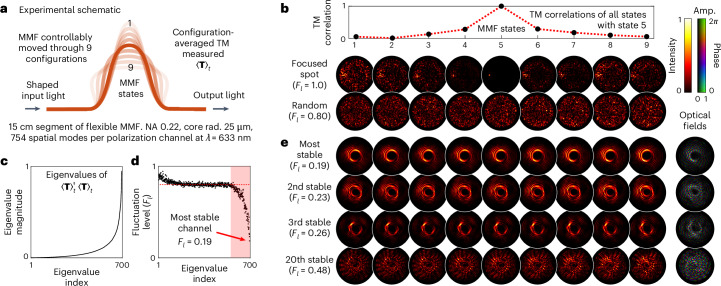
The temporal fluctuation eigenchannels of a bending multimode optical fibre. **a**, Schematic of the experiment. The TM of an MMF is measured in nine different configurations, from which the time-averaged TM 〈**T**〉_*t*_ is calculated. NA, MMF’s numerical aperture; core rad., MMF’s core radius. **b**, Decorrelation of the fibre TM as the MMF is bent. Top: the normalized correlations (absolute value of the overlap integral) between the TM of the MMF in bend state 5 with every other bend state. Supplementary Section 7 shows correlations between all TMs. Middle row: when an output focus is created by calculating an input field based on the TM of the MMF in state 5, we see this focus completely disintegrate as the fibre is bent into other states. Bottom row: a fixed random input field results in different output speckle fields for each bend state, yielding a fluctuation level of *F*_*l*_ = 0.8. **c**, The magnitudes of the eigenvalues of ${\langle {\bf{T}}\rangle }_{t}^{\dagger }{\langle {\bf{T}}\rangle }_{t}$, arranged in ascending order and normalized to a maximum value of 1. **d**, Experimentally measured temporal fluctuation levels when the bending MMF is illuminated with 754 eigenvectors of ${\langle {\bf{T}}\rangle }_{t}^{\dagger }{\langle {\bf{T}}\rangle }_{t}$. The top ~150 channels (region highlighted in pink) exhibit fluctuations lower than that of a random input field (*F*_*l*_ = 0.8, marked with hatched red line). **e**, The transmitted intensity patterns of selected temporal fluctuation eigenchannels as the MMF is moved through all nine bend configurations. Output optical fields (with fibre in bend state 5) are shown to the right.

Figure 4a shows a schematic of our experiment (Methods and Supplementary Section 7). We measure the time-averaged TM of an MMF supporting ~750 spatial modes as it is smoothly moved through 9 different bend configurations. Figure 4b shows how the TM decorrelates as the fibre is bent. Transmitting a fixed random input field through the fibre yields different output speckle patterns for each bend state, corresponding to a temporal fluctuation level of *F*_*l*_ ~0.8.

Figure 4c shows the eigenvalues of matrix ${\langle {\bf{T}}\rangle }_{t}^{\dagger }{\langle {\bf{T}}\rangle }_{t}$. We experimentally measure the temporal fluctuations *F*_*l*_ in the output fields of 754 temporal fluctuation eigenchannels, which are shown in Fig. 4d. We find ~150 channels that exhibit lower fluctuation levels than that of a random input field, across this range of fibre movement. Figure 4e shows examples of the most stable channels—all of which show reduced intensity on the axis (see Supplementary Section 7 for discussion of this phenomenon). See also Supplementary Video 5. This approach may be useful for the transmission of data through MMFs.

## Discussion

We have shown that it is possible to identify and guide light through stable channels within dynamic scattering media. Our methods do not rely on prior knowledge of the location of dynamic regions or type of motion, and only require measurements of externally scattered light made on the same timescale as temporal fluctuations. To understand the potential applications of these techniques, a key question arises: to what extent do such stable channels exist within dynamic scattering media? While there is a large parameter space of possible scattering scenarios, we numerically examine some general trends for random disordered media.

We first use a layered forward scattering model to study how the proportion of stable transmission channels scales as a function of the area fraction of dynamic regions on each layer, and the number of randomly connected layers (that is, medium depth)—see Supplementary Section 11 for details. For the first few layers, we find that the number of stable channels decreases linearly with the dynamic area per layer and the number of layers. However, at greater depths, this trend becomes sub-linear, and a proportion of stable channels are capable of propagating deeply. For example, with a 5% dynamic area ratio per layer, we find that ~10% of the modes remain highly stable to a depth of 20 layers. As with all wavefront shaping approaches, our ability to excite stable channels will depend upon the number of SLM control elements relative to the mode capacity of the medium.

We next consider media in which backscattering is not negligible. Such media pose additional challenges owing to competing requirements: optical fields must both circumnavigate moving regions and also penetrate deeply enough into the medium to transmit substantial levels of power to the other side. In Supplementary Section 13, we explore how stable channels may be identified in this regime. We first show that if we are able to measure the full scattering matrix of a diffusive sample, coherently time-averaging the scattering matrix allows stable channels to be identified. Since the full scattering matrix is difficult to measure in most realistic scenarios, we next show how our physical adjoint optimization approach can be adapted to successfully guide light around dynamic regions while measuring transmitted light only. This modified optimization procedure requires light to be passed back and forth once per iteration on a timescale over which the entire medium is static (the medium is still free to move between iterations). This places more stringent constraints on the update rates of SLMs, yet offers an advantage over conventional wavefront shaping methods in which the medium must remain static throughout the entire optimization process. More broadly, we note that each new choice of adjoint optimization objective function will lead to a new physical optimization procedure, and it will be interesting to further explore this space in the future.

We now evaluate our adjoint methods in the context of entirely dynamic media containing no permanently static regions, but exhibiting multiple decorrelation rates ranging from *τ*_fast_ to *τ*_slow_. If coherent scattered field averaging is carried out over a timescale longer than *τ*_slow_, this average tends to zero and our methods fail. However, we find that as few as two medium realizations will suffice to create useful coherent averages to guide optimizations towards a more stable channel (Supplementary Sections 9, 10 and 12). Thus, if optimization is conducted on a timescale *τ*_opt_ that is *τ*_fast_ < *τ*_opt_ < *τ*_slow_, the resulting channel will preferentially occupy the slowest parts of the medium, gradually losing stability over a timescale of *τ*_slow_—which could be rectified by continuous optimization. Recent work indicates that focusing using conventional wavefront shaping conducted on the same timescale as the medium decorrelation time also preferentially selects for more stable modes^38,39^.

Looking beyond optics, the problem that we have addressed here is closely related to multi-path fading in radio frequency wireless communication channels. In that case, the interaction of transmitted signals with moving media is known as mode-stirring, and the Rician *K*-factor quantifies the ratio of ‘unstirred’ to ‘stirred’ paths^53–56^. Circumnavigation of dynamic regions may potentially be applied at radio and microwave frequencies, either in the spectral domain or in the spatial domain in conjunction with beam-forming systems.

The concepts that we have introduced in this article apply to general wave phenomena and may have relevance to a diverse range of applications. Possibilities include imaging deep into living biological tissue^57–59^, transmission of space-division multiplexed optical communications through turbulent air^60^ and underwater^61^, propagation noise reduction in acoustic beam forming^62^ and emerging smart microwave and radio environments^63^. Our work adds to the toolbox of methods to counteract the adverse effects of dynamic scattering media.

## Methods

### Calculation of temporal fluctuations

Throughout this work, we quantify the degree of temporal fluctuation in the intensity of optical fields transmitted through dynamic scattering media using the real positive scalar *F*_*l*_, which is calculated from a sequence of camera frames recording the transmitted time-dependent intensity patterns in the following way:

4 $${F}_{l}={\bar{\sigma }}_{I}/{\bar{\mu }}_{I},$$

where ${\bar{\sigma }}_{I}$ denotes the standard deviation of the time-fluctuating intensity, averaged over all illuminated camera pixels, and is given by

5 $${\bar{\sigma }}_{I}=\frac{1}{P}\mathop{\sum }\limits_{p=1}^{P}{\left[\frac{1}{T}\mathop{\sum }\limits_{t = 1}^{T}{\left({I}_{p,t}-{\mu }_{p}\right)}^{2}\right]}^{\frac{1}{2}}.$$

${\bar{\mu }}_{I}$ is the mean intensity averaged over all camera pixels at all measured times, given by

6 $${\bar{\mu }}_{I}=\frac{1}{P}\mathop{\sum }\limits_{p=1}^{P}{\mu }_{p},\quad{\mu }_{p}=\frac{1}{T}\mathop{\sum }\limits_{t=1}^{T}{I}_{p,t}.$$

Here *p* indexes the camera pixel and *t* indexes the frame number, so that *I*_*p*,*t*_ is the intensity of the *p*th pixel in the *t*th frame. *P* is the total number of camera pixels, *T* is the total number of frames, and *μ*_*p*_ is the mean intensity detected by camera pixel *p*. This choice of temporal fluctuation quantification ensures that fluctuations are normalized with respect to transmitted power. *F*_*l*_ = 0 for a static transmitted field. *F*_*l*_ = 1 for a series of naturally occurring speckle patterns (that is, exhibiting Rayleigh statistics^64^) that are uncorrelated in time. This second case is derived from the fact that such speckle patterns have a negative-exponential intensity probability distribution function, where the probability of a point in space having intensity *I* is given by ${P}_{r}(I)=\left(1/\bar{I}\right)\exp \left(I/\bar{I}\right)$, and here $\bar{I}$ is the mean intensity of the speckle pattern. The standard deviation and mean of this distribution are equal; thus, *F*_*l*_ = 1 for a sequence of uncorrelated speckle patterns.

### Unguided optimization

The optimization commences by transmitting an initial trial field **u**_0_ through the sample and recording a series of transmitted intensity images with the camera. We sample 20 realizations of the fluctuating speckle pattern and calculate the level of temporal fluctuations *F*_*l*_ over these frames. In this initial experiment, we looped the same 20 randomly generated phase patterns inside the patches on each iteration. The input SLM used to generate the incident fields is subdivided into 1,200 super-pixels. The phase delays imparted by these super-pixels represent the independent degrees of freedom that we aim to optimize. We begin by setting each super-pixel to a random phase value, creating incident field **u**_0_, and measure the level of output fluctuations. Next, two new test fields are sequentially transmitted through the sample. These are generated by randomly selecting half of the input SLM super-pixels used to create **u**_0_, and adding/subtracting a small constant phase offset *δθ* from these pixels, yielding inputs **u**_±*δθ*_. Here we used *δθ* = *π*/40. We measure the corresponding level of output fluctuations for these two new trial inputs, and if either exhibit lower fluctuations than **u**_0_, the optimized input field is updated accordingly. This process is repeated until the output fluctuation level no longer improves.

This algorithm relies on accurately capturing the output fluctuations on each iteration. However, there is an uncertainty in the measurement of *F*_*l*_ owing to the finite number of realizations of the dynamic medium sampled and the presence of other sources of noise. To enhance the algorithm’s robustness to this source of noise, on each new iteration, the optimum input field from the last iteration is retested and compared with the two new trial fields—doing so increases the optimization time, but crucially prevents a single measurement with an erroneously low value of *F*_*l*_ from blocking the optimizer from taking steps in subsequent iterations.

### Physical adjoint optimization

To increase *C*, the input field at iteration *i* + 1 is given by **u**^(*i*+1)^ = **u**^(*i*)^ + *δ***u**. Our task is to find the elements of column vector *δ***u** at each iteration. The *j*th element of *δ***u** is given by $\delta {u}_{j}=\delta A{{\rm{e}}}^{i{\theta }_{j}}$. Here *δA* is the optimization step size, which is set to the same value of a small positive constant for all elements of *δ***u**. *θ*_*j*_ is the phase change of element *j*, which in general will be different for all elements of *δ***u**. All of these phase changes components can be found simultaneously (see Supplementary Section 3 for derivation), which when stacked in column vector ***θ*** are given by

7 $${\boldsymbol{\theta }}=-\arg \left({{\bf{T}}}^{T}\cdot {\langle {\bf{v}}\rangle }_{t}^{* }\right),$$

where ${\langle {\bf{v}}\rangle }_{t}^{* }$ is the phase conjugate of the time-averaged output field.

As shown in Fig. 2a, iteration *i* commences by illuminating the dynamic scattering medium from the left-hand side (LHS) with trial field **u**^(*i*)^, and time-averaging the transmitted optical field on the right-hand side (RHS), yielding 〈**v**〉_*t*_. Equation (7) specifies that 〈**v**〉_*t*_ should be phase conjugated and transmitted in the reverse direction through the dynamic medium, from the RHS back to the LHS. Measuring the phase of the resulting field on the LHS reveals how to update all spatial components of the input field to improve *C*, generating the next input **u**^(*i*+1)^.

Experimentally, this adjoint field optimization strategy requires a relatively complicated optical set-up: two digital optical phase conjugation (DOPC) systems—which enable time reversal of optical fields—are arranged back-to-back on either side of the dynamic sample. We use single-shot off-axis digital holography to measure the output fields on each side. The DOPC systems require very precise alignment, so we implemented a calibration method that we recently described in ref. ^65^. Our set-up enables spatial shaping of both the intensity and phase profile of the time-reversed field travelling in both directions. We test this approach to guide light through a similar sample dynamic medium to that used for unguided optimization (Fig. 1e, top row). To construct the time-averaged field transmitted through the medium, we coherently average the field scattered through *N* = 5 different realizations of the dynamic parts of the medium. Supplementary Section 4 shows a schematic of the full optical set-up used in this experiment.

We note that, in principle, adjoint optimization could be used to find multiple stable fields—by conducting a series of adjoint optimizations, each seeded from a different initial field. This would lead to a set of stable output fields that can be stored as the column vectors of matrix **V**, and used to generate a target output field **v**_trg_ by injecting into the medium the field **u** = **V**^−1^**v**_trg_. However, this is not an efficient search strategy, since there is no way to guarantee the linear independence of the set of optimized fields—meaning that very similar fields may be inadvertently found. Hence, we developed the final approach based on the time-averaged TM.

### Temporal fluctuation eigenchannels of the time-averaged TM

For the experiments shown in Fig. 3, we illuminate the sample with *M* = 2,304 probe fields, and average the output field sampling *N* = 10 uncorrelated realizations of the scattering medium for each input mode. Supplementary Section 5 describes the full optical set-up for this experiment. For the experiments shown in Fig. 4, we illuminate the MMF with *M* = 1,600 probe fields and measure its TM in 9 different bend states. Supplementary Section 7 describes the full optical set-up for this experiment. Experimentally, measurement of the time-averaged TM is simpler than physical adjoint optimization—although the main challenge is that the reference beam required for holographic field measurement must be phase-drift-stabilized for the entire measurement of **T**_av_.

## Online content

Any methods, additional references, Nature Portfolio reporting summaries, source data, extended data, supplementary information, acknowledgements, peer review information; details of author contributions and competing interests; and statements of data and code availability are available at 10.1038/s41566-025-01642-z.

## Supplementary information

Supplementary Information Supplementary Sections 1–14, which include Figs. 1–22.

Supplementary Video 1 Camera frames showing the time-dependent intensity profile of light transmitted through a dynamic scattering medium. Left panel: initial field. Right panel: optimized field, found using unguided optimization. We observe that the optimized field exhibits much lower levels of time-dependent fluctuations than the initial field. Scale bar shows relative intensity.

Supplementary Video 2 Camera frames showing the time-dependent intensity profile of light transmitted through a dynamic scattering medium. Left panel: initial field. Right panel: optimized field, found using physical adjoint optimization. We observe that the optimized field exhibits much lower levels of time-dependent fluctuations than the initial field. Scale bar shows relative intensity.

Supplementary Video 3 Camera frames showing the time-dependent intensity profile of light transmitted through a dynamic scattering medium. Each panel shows the transmitted light when a particular fluctuation eigenchannel is excited (the eigenchannel index is labelled above each panel). We observe that the light transmitted through the high-index eigenchannels fluctuates the least, while light transmitted through low-index eigenchannels exhibits much higher levels of temporal fluctuations. Scale bar shows relative intensity.

Supplementary Video 4 Camera frames showing the time-dependent intensity profile of light transmitted through a dynamic scattering medium. Left panel: the result of attempting to form a focus at the output using the conventional inverse TM. Right panel: the focus generated through the same sample, formed by exciting only the top 100 most stable fluctuation eigenchannels. In both cases, we scan the position of the focus over four different positions. We observe that the focus created using the stable eigenchannels is enhanced: it has a higher contrast and exhibits lower levels of temporal fluctuations. Scale bar shows relative intensity.

Supplementary Video 5 Camera frames showing the time-dependent intensity fluctuations of the most stable fluctuation eigenchannels projected through the MMF as it is moved through its nine configurations (top row). The bottom row shows the fluctuations of unstable eigenchannels (left two panels) and random inputs (right two panels).

## Data Availability

The data supporting the findings of this study are available within the paper, its Supplementary Information files and at the University of Exeter data repository^66^.
