# Supplementary material for: Threading light through dynamic complex media
Source: Nat Photonics. 2025 Mar 14;19(4):434–40. doi: 10.1038/s41566-025-01642-z (PMC11968404; doi:10.1038/s41566-025-01642-z)
Supplement: Supplementary file 1 — Supplementary Sections 1–14, which include Figs. 1–22. [file 41566_2025_1642_MOESM1_ESM.pdf]

nature photonics

Supplementary information

https://doi.org/10.1038/s41566-025-01642-z

Threading light through dynamic complex media

In the format provided by the authors and unedited

CONTENTS

§1: **Unguided optimisation – experimental details**

§2: **Numerical reconstruction of the fields at each phase plane inside the emulated dynamic scatterer**

§3: **Physical adjoint optimisation – derivation of method**

§4: **Physical adjoint optimisation – experimental details**

§5: **Time-averaged transmission matrix – experimental details**

§6: **Focusing through dynamic scattering media using the time-averaged transmission matrix**

§7: **Time-averaged TM of a bending multimode optical fibre – experimental details**

§8: **Numerical simulations of experiments and noise considerations**

§9: **Number of medium realisations per iteration required for physical adjoint optimisation**

§10: **Physical adjoint optimisation with high levels of movement**

§11: **Number of stable channels through lossless dynamic media**

§12: **Number of medium realisations needed to find stable channels using the time-averaged TM**

§13: **Exploration of different scattering regimes**

§14: **Description of supplementary movies**

§1: Unguided optimisation – experimental details

Figure 1 shows a schematic of the experimental setup used to test our unguided optimisation strategy. In summary: we emulate a dynamic scattering medium using a cascade of 3 phase planes, implemented using 3 reflections from a liquid crystal SLM (SLM_2) with a mirror placed parallel to and facing the SLM chip. It is important to calibrate the voltage-to-phase lookup table of this SLM to ensure that the correct phase delays are imparted to reflected light, and that a 2π rad phase range is achievable. The beam incident on the scattering medium is shaped using SLM_1, and a camera records the fluctuations of the output field. In this experiment the camera is synchronised with (and triggered by) SLM_2, so pictures are always taken at the same point in the update cycle to minimise spurious intensity fluctuations caused by SLM update flicker (also see §8 for more discussion of this effect).

We now describe this setup in more detail: An optical beam emanating from a continuous wave laser (wavelength 632.8 nm, power 21 mW, linearly polarized) is expanded using lenses L_1-L_2 and coupled into a polarization maintaining fiber (PMF) using a fiber collimator (FC). The optical power entering the system is adjusted using a combination of a half wave-plate (HWP_1) and a polarizing beam-splitter (PBS_1). The optical beam diverging from other end of the fiber is collimated using lens L_3. The beam's optical power and polarization is controlled using the combination of half-wave-plates (HWP_2 - HWP_3) and polarizing beam-splitter (PBS_2). The beam is then expanded using lenses L_4-L_5 to overfill the active area of SLM_1 – used to shape the light incident on the dynamic scattering medium. SLM_1 imparts a spatially varying phase delay to the incident beam, and is also encoded with a phase grating to diffract the desired optical field into the 1st diffraction order. Light diffracted into other unwanted diffraction orders is blocked out by an iris (IR_1). The spatially filtered beam is re-imaged using lenses L_7-L_8 to the dynamic scattering medium, emulated by SLM_2. The beam sequentially reflects from three separate regions of SLM_2. The outgoing beam is then re-imaged using lenses L_9-L_{10} to the camera.

The unguided optimisation proceeds as follows: SLM_1 is subdivided into 1200 equally sized square super-pixels. Initially, each superpixel imparts a random phase to incident light. Our aim is to optimise the phase of these super-pixels such that the transmitted field circumnavigates any dynamic regions of the sample, which is emulated using SLM_2. SLM_2 is set to display a static smoothly varying phase pattern with three square patches (of size $\sim 20 \times 20$ SLM pixels) inside which the phase dynamically varies. The location of these three patches each coincide with a different reflection area of the light propagating through the

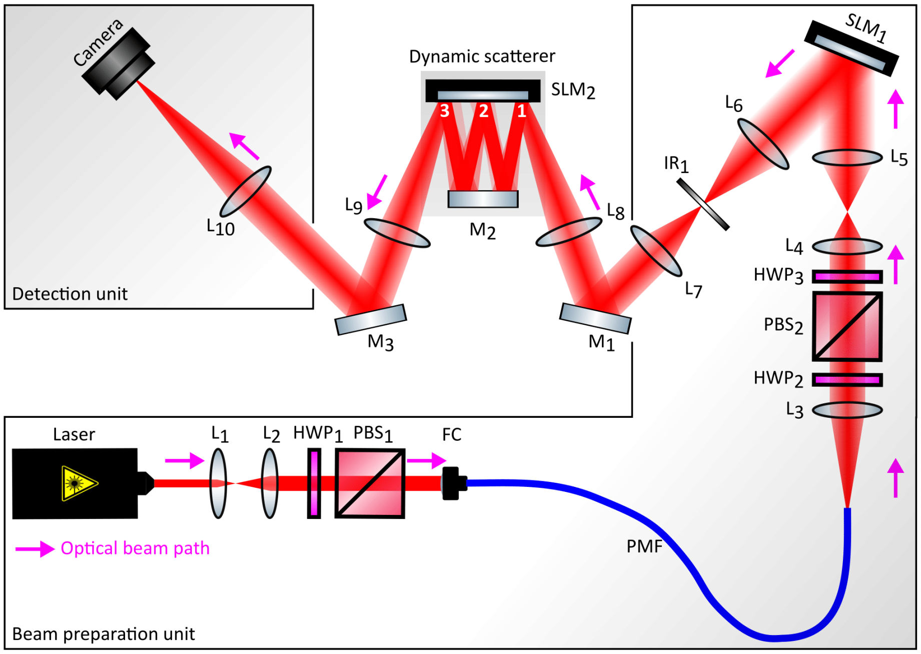

Figure 1. **Experimental setup for unguided optimization**. Component information: Laser: Thorlabs-HNL210L. SLM_1: Hamamatsu LCOS 800×600 pixel LC-SLM. SLM_2: Holoeye Pluto-2 1920×1080 pixel LC-SLM (with dielectric backplane optimised for high efficiency reflection at 633 nm wavelength). Lenses: L_1 (focal length f = 50 mm), L_2 (f = 100 mm), L_3 (f = 60 mm), L_4 (f = 150 mm), L_5, L_6 (f = 300 mm), L_7 (f = 200 mm), L_8 (f = 150 mm), L_9 (f = 250 mm), L_{10} (f = 200 mm)). Mirrors: M_1, M_3 are 1" mirrors, M_2 = 10 mm square mirror (Edmund optics). PMF = polarization maintaining fiber (Thorlabs, P3-630PM-FC-1, length 1 m). FC = fiber collimator: Thorlabs F110APC-633. HWP = Half wave-plate. PBS = Polarizing beamsplitter. IR = iris. Camera: Basler piA640-210gm. We thank A. Frazer for use of the inkscape component library.

SLM-mirror system. SLM_2 updates at a rate of 20 Hz and cycles through a series of pre-saved patterns as the camera records 100 frames, from which level of fluctuations are calculated. This procedure is repeated for a second test input field, for which 600 randomly chosen super-pixels have their phase uniformly modulated by +pi/40 rad. Finally, this procedure is repeated for a third test input field, for which the same 600 randomly chosen super-pixels have their phase uniformly modulated by -pi/40 rad. The incident field which corresponds to the lowest level of fluctuations is used as the starting field for the next iteration of the optimisation. We run our optimisation until the level of fluctuations no longer reduce, which in this case was after about 3000 iterations.

§2: **Numerical reconstruction of the fields at each phase plane inside the emulated dynamic scatterer**
As we have full control over the test scattering medium, we are able to digitally 'peel back' the outer scattering layers to look inside and directly observe the evolution of the optimised field as it propagates through the cascade of phase planes. Experimentally this is achieved by switching-off the aberrating effect of the outer planes, and imaging the optimised field that is incident on plane 2. We recover the phase of this optical field using phase-stepping full-field holography, see e.g. ref. [9]. The coherent reference needed for this measurement is obtained by splitting off part of the laser beam at PBS_2. SLM_1 is then used to globally phase shift the incident field with respect to the reference, enabling measurement of the field at plane 2 by imaging it along with the coherent reference beam, onto the camera. We measure the field at plane 2 twice: firstly with the phase profile of planes 2 and 3 switched off (i.e. set to uniform 0 everywhere) – yielding the field $\mathbf{q}_1$ and secondly with only the phase profile of plane 3 switched off – yielding $\mathbf{q}_2$, which includes the effect of the second phase plane. $\mathbf{q}_1$ and $\mathbf{q}_1$ essentially represent the field immediately before and immediately after the second phase plane. Numerically back-propagating $\mathbf{q}_1$ a distance δz yields a reconstruction of $\mathbf{q}_0$ – the optimised field just after plane 1. The numerical propagation is carried out using the angular spectrum method. Numerically forward-propagating $\mathbf{q}_2$ through free-space a distance δz yields $\mathbf{q}_3$ – the optimised field just before plane 3. The fields adjacent to each plane, $\mathbf{q}_0$, $\mathbf{q}_1$ and $\mathbf{q}_3$ are the three fields shown in Fig. 1(e) second row.

§3: **Physical adjoint optimisation – derivation of method**
Here we give a full derivation of the physical adjoint optimisation method, which is discussed also below in Sec. 8. Consider a 2D system of randomly arranged scatterers placed between a source plane and a detector plane. The source field is given by $u(x)$, and the field at the detectors is given by $v_t(x)$. We can then write the field on the detection plane due to the configuration of particles at time t

$$v_t(x) = \int G_t(x, x')u(x')dx', \tag{1}$$

where the integral over the Green's function G_t for the configuration of particles at time t is equivalent to multiplying the transmission matrix $\mathbf{T}$ by the source field - the notation used in the main body of this paper. To minimise the fluctuations in the transmitted field, we aim to maximise the overlap between the output modes at all times t, given by $\mathcal{F}_1$:

$$\mathcal{F}_1 = \left| \frac{1}{N^2} \sum_t \sum_{t'} \int dx\; v_t(x)\, v^*_{t'}(x) \right|^2 . \tag{2}$$

As discussed in Sec. 8, maximizing $\mathcal{F}_1$ is also equivalent to maximizing the norm of the time–averaged field on the output plane. In order to understand how to iteratively maximise $\mathcal{F}_1$, we consider a small change in the output field of δv_t:

$$\begin{aligned} \mathcal{F}_1 &= \frac{1}{N^2} \left| \sum_t \sum_{t'} \int dx\; [v_t + \delta v_t]\,[v^*_{t'} + \delta v^*_{t'}] \right|^2 \\ &= \frac{1}{N^2} \left| \sum_t \sum_{t'} \int dx\; [v_t\, v^*_{t'} + v_t\, \delta v^*_{t'} + \delta v_t v^*_{t'} + {\color{red}\delta v_t \delta v^*_{t'}}] \right|^2 , \end{aligned} \tag{3}$$

Being second order in smallness, we drop the red term since it is the product of two small numbers and so can be considered negligible. Expanding Eq. (3) to leading order in the small change in the field δv_t,

$$\begin{aligned} \mathcal{F}_1 &= \frac{1}{N^2} \left| \sum_t \sum_{t'} \int dx\, v_t\, v^*_{t'} + 2 \sum_t \sum_{t'} \int dx\, \mathrm{Re}\,[v^*_{t'} \delta v_t] \right|^2 \\ &= \left| \int dx\, |\langle v \rangle|^2 + \frac{2}{N} \sum_t \int dx\, \mathrm{Re}\,[\langle v \rangle^* \delta v_t] \right|^2 . \end{aligned} \tag{4}$$

where we have used the expression for the average field $\langle v \rangle = N^{-1}\sum_t v_t$. At this point we use the relationship between input and output fields (1) to write the small change in the transmitted field in terms of a small change in the input field, $\delta u(x')$ (which we note does not depend on time)

$$\delta v_t\,(x) = \int dx'\; G_t\,(x,x')\,\delta u(x'). \tag{5}$$

Applying this representation of the change in the field to Eq. (4), the figure of merit can now be written in terms of the change in the input field $\delta\mathbf{u}$, over which we have control

$$\mathcal{F}_1 = \left|\int dx\,|\langle v(x)\rangle|^2 + \frac{2}{N}\sum_t\int dx\,\mathrm{Re}\left[\langle v(x)\rangle^*\int dx'\;G_t\,(x,x')\,\delta u(x')\right]\right|^2 \tag{6}$$

As the system is reciprocal $G_t(x,x') = G_t(x',x)$, we can rewrite Eq. (6) as

$$\mathcal{F}_1 = \left|\int dx\,|\langle v(x)\rangle|^2 + \frac{2}{N}\,\mathrm{Re}\left[\int dx'\delta u(x')\sum_t\int dx\,G_t\,(x',x)\,\langle v(x)\rangle^*\right]\right|^2 \tag{7}$$

Given that the first term inside the absolute value on the right of Eq. (7) is positive, to increase the value of $\mathcal{F}_1$ through the change to the input, δu, the real value taken in Eq. (7) must be positive. There are various ways to achieve this. In general the change to the input field yielding the maximal increase in $\mathcal{F}_1$ must take the form

$$\delta u(x') = \delta A(x')\mathrm{e}^{i\theta(x')}, \tag{8}$$

where the amplitude δA is an arbitrary positive quantity, and the phase is

$$\theta = -\mathrm{arg}\left[\sum_t\int G_t(x',x)\,\langle v^*(x)\rangle\,dx\right]. \tag{9}$$

This choice ensures that the quantity appearing within the real part on the right of Eq. (7) is positive and real. The simplest way to enforce the condition of maximal increase (8) is to change the input field in proportion to the conjugate of $\sum_t\int G_t(x',x)\,\langle v^*(x)\rangle\,d^3x$,

$$\delta u(x') = \eta\left[\sum_t\int G_t(x',x)\,\langle v^*(x)\rangle\,dx\right]^*, \tag{10}$$

where η is a positive normalisation constant we choose to ensure that the change in the input field is small. Eq. (10) shows that we can increase the figure of merit (1) through sending a given input field through the system, averaging over time, sending this average field back to the input plane and then averaging for a second time. Taking the complex conjugate of this final average and adding this to the input field increases the figure of merit $\mathcal{F}_1$.

The result of applying this alternative iterative procedure is that the input field approaches the largest singular vector of the average transmission matrix (here $N^{-1}\sum_t G_t(x,x')$). We can see this through re–writing Eq. (10) in terms of the initial field u

$$\delta u(x') = \eta\int dx\int dx''\langle G^\star(x',x)\rangle\langle G(x,x'')\rangle u(x'') \equiv \eta\langle\mathbf{T}\rangle^\dagger\cdot\langle\mathbf{T}\rangle\cdot\boldsymbol{u}, \tag{11}$$

where we have adopted a vector notation for the field and the time averaged transmission matrix in the final step. After $\mathcal{N}$ iterations of this process the input field thus becomes

$$\boldsymbol{u}_{\mathcal{N}} = \left(\mathbf{1}+\eta\langle\mathbf{T}\rangle^\dagger\cdot\langle\mathbf{T}\rangle\right)^{\mathcal{N}}\cdot\boldsymbol{u}_0 \tag{12}$$

where $\boldsymbol{u}_0$ is the initial input field. The largest eigenvalue of the matrix $\mathbf{1}+\eta\langle\mathbf{T}\rangle^\dagger\cdot\langle\mathbf{T}\rangle$ equals $1+\eta\sigma^2_{\mathrm{max}}$, where σ_{max} is the largest singular value of the average transmission matrix. Therefore with increasing $\mathcal{N}$, the input field (12) converges to the singular vector of $\langle\mathbf{T}\rangle$ with the largest singular value.

We note that the time-averaged transmission matrix generally reduces the norm of an input field, making a unitary matrix at any time instant non–unitary once time averaged, with eigenvalues less than or equal to unity. This is similar to the effect of additional inhomogeneous absorption in the system, where the norm of the output field is generally less than the input.

Just as the largest singular values of a transmission matrix in an absorbing system represent the fields that avoid absorbing regions [4, 5], here these fields avoid regions causing maximum fluctuation in the output.

§4: **Physical adjoint optimisation – experimental details**

Figure 2 shows a schematic of the experimental setup used to test the physical adjoint optimisation strategy. The dynamic scattering medium is created in the same way as described above, using 3 reflections from an SLM.

The dynamic medium is first illuminated from the left hand side, as shown in the upper schematic in Fig. 2. The laser (same as detailed above) is expanded and half-wave-plate HWP_1 is set so that light reflects at beam-splitter PBS_1. The beam is split into a signal and reference arm using polarising beam-splitter PBS_3. The signal beam is shaped by SLM_3, which in our experiment is a digital micro-mirror device (DMD). Here we implement intensity and phase beam shaping using the DMD as described in [7], and iris IR_3 blocks the unwanted diffraction orders. The shaped beam passes through the dynamic medium (SLM_2) to camera 1. The reference beam is directed around the dynamic scatterer to camera 1, and the interference of the two beams enables the time-evolving scattered field to be measured using single-shot off-axis digital holography. The time-averaged optical field is calculated from 5 optical fields – this number chosen empirically after testing different averaging times. During this phase of the experiment, SLM_1 (an LC-SLM) performs no beam shaping, but displays a uniform grating to direct light through iris IR_1.

Light is next sent through the system in the opposite direction, as shown in the lower schematic in Fig. 2. To switch the laser direction, half-wave-plate HWP_1 is rotated so light transmits through beam-splitter PBS_1. If necessary, this step could be automated for fast operation in the future. The beam is then split into a signal and reference arm using polarising beam splitter PBS_2. The signal beam is now shaped by SLM_1, to create the phase conjugate of the time-averaged optical field measured in the previous step. Here we implement intensity and phase beam shaping using the phase-only SLM as described in [6], and iris IR_2 blocks the unwanted diffraction orders. The beam passes through the dynamic medium (SLM_2) to camera 2. The reference beam is directed around the dynamic scatterer to camera 2, and the interference of the two beams once again enables the time-evolving scattered field to be measured using single-shot off-axis digital holography. The time-averaged optical field, and consequently the updated input field, are calculated. During this phase of the experiment, SLM_3 performs no beam shaping, but displays a uniform grating to direct light through iris IR_4. This completes one iteration of our physical adjoint optimisation strategy, which is then repeated until the optimised field converges. We note that the correct alignment of the digital optical phase conjugation systems on either side of the sample is critical to the performance of the system. Our alignment procedure is explained in detail in ref. [8]. Once the optimisation is complete, Camera 3 is used to observe the propagation of the optimised beam inside the medium.

§5: **Time-averaged transmission matrix – experimental details**

Figure 3 shows a schematic of the experimental setup used to measure the time-averaged TM. The laser beam is split into a signal and reference path at PBS_2. The light in the signal beam path is shaped by SLM_1, and passes through the dynamic scattering medium to the camera, where it interferes with light from the reference beam path. A sequence of 2304 orthogonal input probe fields are transmitted through the medium, and the output field is measured using single-shot off-axis digital holography. The output field is averaged over 10 configurations of the dynamic medium for each probe mode. It is critical that the phase of the reference beam does not drift with respect to the light in the signal arm throughout this measurement. A standard approach to negate the effect of phase drift during TM measurement is to interlace the changing input probe measurements with the projection of a standard probe field, the mode of which is held constant throughout the measurement. The global phase of this probe field tracks the phase drift between the arms of the interferometer, which can then be subtracted. Here, we found that this method can still be applied to a partially moving sample – as long as the sample fluctuations are minor, the global phase of a standard probe mode faithfully tracks the phase drift. Ideally the probe mode would itself be stable, and so in some cases it may be necessary to first optimise a single stable probe mode to use for drift tracking, before measuring the time-averaged TM. In our experiments we found such a pre-optimisation step to be unnecessary.

§6: **Focusing through dynamic scattering media using the time-averaged transmission matrix**

In order to use the information held in the time-averaged TM for stable beam shaping through dynamic scattering media we first calculate the eigenvectors of matrix $\mathbf{T}_{\text{av}}^{\dagger}\mathbf{T}_{\text{av}}$, yielding: $\mathbf{T}_{\text{av}}^{\dagger}\mathbf{T}_{\text{av}} = \mathbf{U}\Lambda\mathbf{U}^{\dagger}$, where as is convention, $\mathbf{U}$ is a matrix of eigenvectors held on its columns, and Λ a diagonal matrix of eigenvalues. We build a new matrix $\mathbf{U}'$ that retains only the columns of $\mathbf{U}$ that have associated eigenvalues with absolute values greater than a stipulated threshold level. The number of eigenvalues above the threshold counts the number of independent fields that are able to thread around any moving regions of the medium. Therefore we can recover a static transmission matrix $\mathbf{V} = \mathbf{T}\mathbf{U}'$ and use it to generate a target output field $\mathbf{v}_{\text{trg}}$ by injecting $\mathbf{u} = \mathbf{U}'\mathbf{V}^{\dagger}\mathbf{v}_{\text{trg}}$ (where here we have taken the conjugate transpose as an approximation to the inverse of $\mathbf{V}$). Here the input field $\mathbf{u}$ and output field $\mathbf{v}_{\text{trg}}$ are represented in the input and output bases originally used to measure the time-averaged TM. Note that when defining matrix $\mathbf{V}$, then matrix $\mathbf{T}$ can be any of the previously measured TMs $\mathbf{T}(t)$, or the average TM $\mathbf{T}_{\text{av}}$, since the fields described by $\mathbf{V}$ should not impinge on the moving regions of the complex medium.

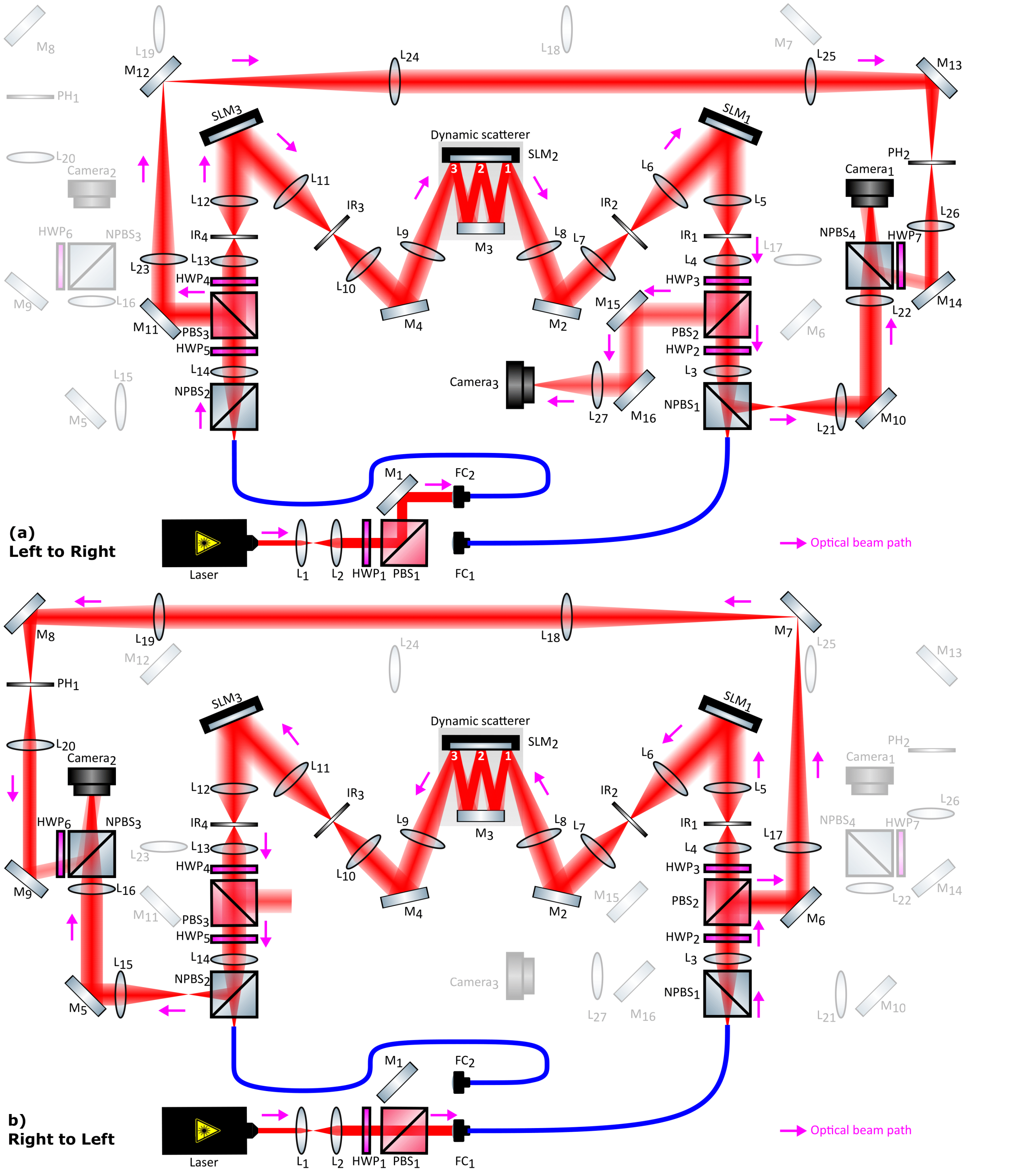

Figure 2. **Experimental setup for physical adjoint optimization**. (a) Configuration for light to be sent through the scattering medium from left to right. (b) Configuration for light to be sent through the scattering medium from right to left. Description of the components: Laser: Thorlabs-HNL210L. SLM$_1$ and SLM$_2$: Holoeye Pluto 2 LC-SLM. SLM$_3$: Vialux V-7001 DMD. Lenses: L$_1$ (f = 50 mm), L$_2$ (f = 100 mm), L$_3$, (f = 60 mm), L$_4$, (f = 150 mm), L$_5$, L$_6$ (f = 300 mm), L$_7$ (f = 200 mm), L$_8$ (f = 150 mm), L$_9$ (f = 150 mm), L$_{10}$ (f = 200 mm), L$_{11}$ (f = 300 mm), L$_{12}$ (f = 300 mm), L$_{13}$ (f=150 mm), L$_{14}$ (f = 60 mm) , L$_{15}$ (f = 35 mm), L$_{16}$ (f = 200 mm)), L$_{17}$ (f=300 mm) and L$_{18}$ (f = 300 mm); L$_{19}$ and L$_{20}$. PMF = polarization maintaining fiber: Thorlabs, P3-630PM-FC-1, length 1 m. HWP = Half wave-plate. PBS = polarizing beam splitter. NPBS = Non-polarizing beam splitter. IR = iris. PH = Pinhole. Cameras 1,2: basler piA640-210gm, 640 $\times$ 480 pixels. Camera 3: Thorlabs Thorcam DCC1545M-GL.

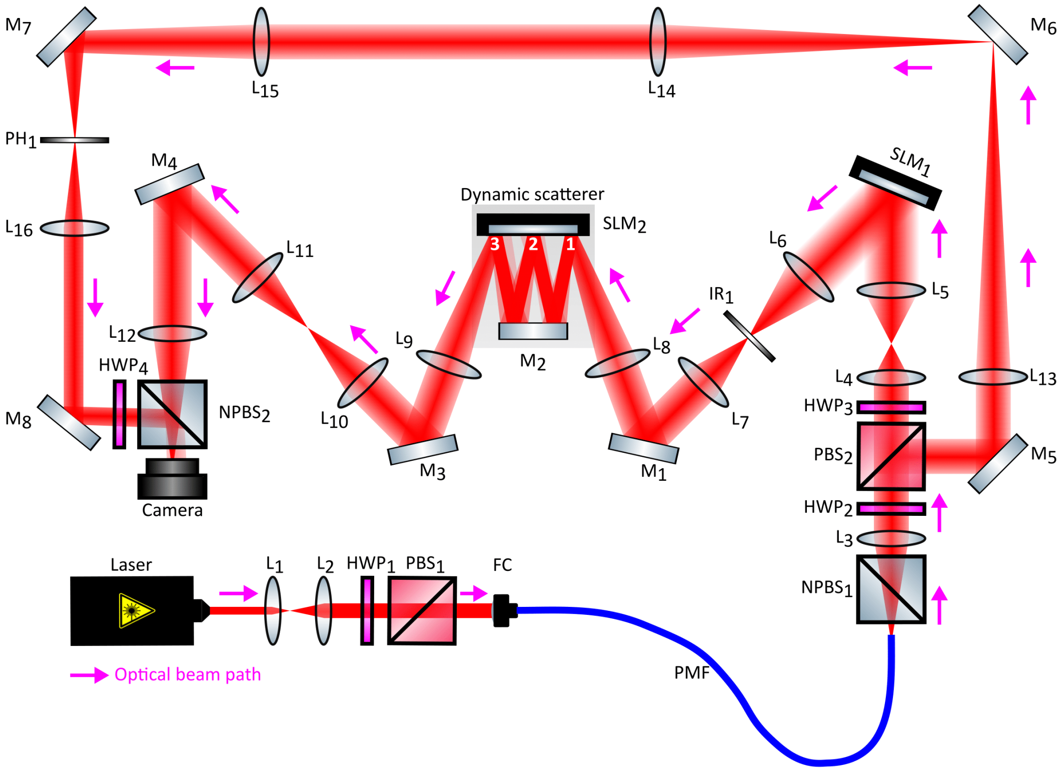

Figure 3. **Experimental setup for time-averaged transmission matrix measurement**. Description of the components: Laser: Thorlabs-HNL210L. SLM_1, SLM_2: Holoeye Pluto 2 LC-SLM. Lenses: L_1 (f = 50 mm), L_2 (f = 100 mm), L_3, (f = 60 mm), L_4, (f = 150 mm), L_5, L_6 (f = 300 mm), L_7 (f = 200 mm), L_8 (f = 150 mm), L_9 (f = 150 mm), L_{10} (f = 200 mm), L_{11} (f=75 mm), L_{12} (f=175 mm), L_{13} (f=300 mm), L_{14} (f = 300 mm), L_{15} (f = 100 mm), L_{16} (f = 50 mm)). PMF = polarization maintaining fiber: Thorlabs, P3-630PM-FC-1, length 1 m). HWP = Half wave-plate. PBS = polarizing beam splitter. NPBS = Non-polarizing beam splitter. IR = iris. PH = Pinhole. Camera: basler piA640-210gm, 640 × 480 pixels.

§7: **Time-averaged TM of a bending multimode optical fibre – experimental details**

Figure 4 shows a schematic of the experimental setup used to measure the time-averaged TM of a multi-mode optical fibre. The laser beam is split into a signal and reference path at PBS1. The light from the signal arm is shaped by the DMD, which is placed in the Fourier plane of the proximal facet of the multi-mode fibre. We first characterize DMD induced aberrations using in-situ aberration correction in the super-pixel basis onto Camera 0, from the back-reflections from the proximal facet of the multi-mode fibre, following the methods described in [11]. From these measurements, we calculate a DMD hologram (ViALUX V-7001) to generate and scan focused spots across the proximal facet of the MMF – these form the basis on input modes used to measure the TM of the MMF. The step-index MMF (ThorLabs FG050UGA) is approximately 50 cm long with a numerical aperture of 0.22 and a core radius $a = 25$ µm. It thus supports approximately 754 spatial modes per polarization channel.

To measure the TM of the MMF, a 40×40 Cartesian grid of focused spots is sequentially projected onto the proximal facet of the multi-mode fibre, with the disk representing the core fitting inside this grid. The distal facet of the fibre is re-imaged onto Camera 1 (Basler piA640-210gm) where the transmitted light interferes with the reference beam that has been guided around the MMF through a polarisation maintaining single mode fibre. Phase stepping holography, with 4 phase steps, is used to reconstruct the phase profile of each output field. We also employ drift correction to remove phase drift between the signal and reference arms of the interferometer: after one set of phase steps, a known phase reference mode is projected through the system and its global phase measured. This provides a measure of how the phase drift evolves through the TM measurement, which can then be corrected. Each TM hence maintains a self-consistent global phase across the columns of the TM. The amplitude of each output field is calculated directly from the square root of the intensity image on the camera (with the light from the external reference arm blocked). When measuring these intensities, we subtract a value according to the noise floor of 2 from each pixel on the camera (8-bit depth, maximum value 255) before constructing the amplitudes. Any optical field from outside the core-cladding boundary is removed numerically, on both facets (i.e., by deleting the appropriate rows and the columns of the TM). Any non-zero fields outside the numerical aperture of the distal facet are also numerically removed.

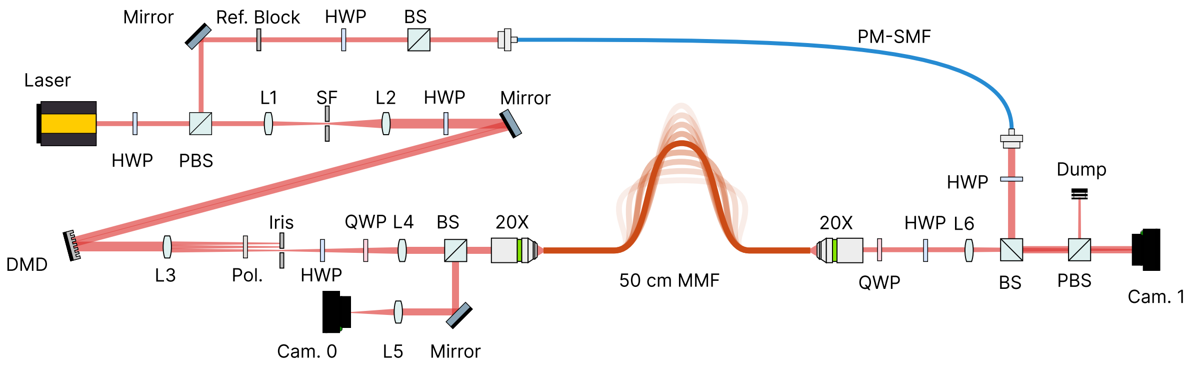

Figure 4. **Experimental setup for time-averaged transmission matrix measurement of a multi-mode fibre**. Description of the components: Laser: Thorlabs-HNL210L 27 mW, 633 nm. DMD: ViALUX V-7001 DMD. Lenses: L_1 (f = 50 mm), L_2 (f = 400 mm), L_3 (f = 300 mm), L_4 (f = 200 mm), L_5, & L_6 (f = 200 mm). PMF = polarization-maintaining fiber: Thorlabs, P3-630PM-FC-1, length 1 m). QWP = quarter wave-plate. HWP = half wave-plate. PBS = polarizing beam splitter. BS = non-polarizing beam splitter. Pol. = linear polarizer. SF = 50 µm spatial filter. Camera 0, 1: basler piA640-210gm, 640 × 480 pixels. 20× refers to an Olympus PLN-20X objective. The Ref. block can be switched into place allowing the external reference path to be blocked when measuring the transmitted signal through the MMF, to measure the amplitude of each transmitted mode.

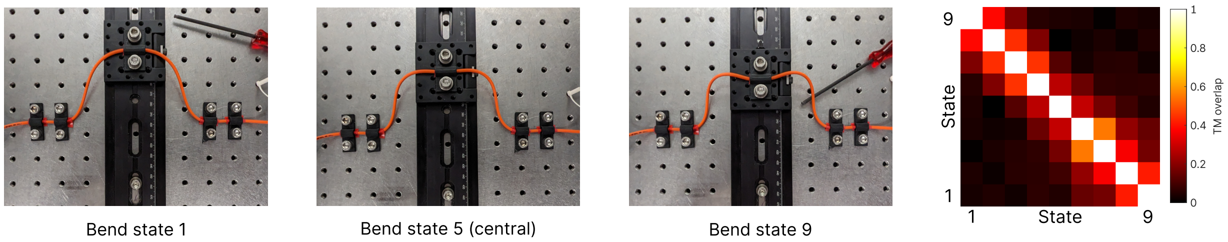

Figure 5. The straight portions of the step-index multi-mode fibre were mounted securely to the optical table. The central part, the flexible portion, was clamped to a linear stage in the centre to change the configuration repeatably. $\sim$ 7.5 cm of fibre on either side of the clamp is allowed to flex (see Fig. 5). The most extreme bend states (1, 9) are shown, along with the central bend state (5). The total travel was 20 mm along the linear stage, with 2.5 mm intervals. The TM was measured in each configuration to determine the time-averaged TM. To ensure that the TM measured in each configuration is distinct (i.e., no single measured TM can approximate all bend states), we take the amplitude of the normalized inner product of each measured TM with every measured TM and plot the result in a confusion matrix.

The TM of the MMF is measured 9 times – while the fibre is positioned in 9 different configuration states. Examples of the configurations that the MMF was moved through are shown in Fig. 5. To find the time-averaged TM, the global phase difference θ_t, between each of the TMs must be found (here t indexes the TMs from 1 to 9). This is achieved by measuring the phase of the overlap integral between the first measured TM, $\mathbf{T}_1$ and all of the other TMs:

$$\theta_t = \arg\left(\sum \mathbf{T}_t \circ \mathbf{T}_1^*\right), \tag{13}$$

where $\mathbf{T}^*$ is the complex conjugate of $\mathbf{T}$, $\circ$ is the element-wise product between the two matrices, and the sum is over all TM elements. Hence the time averaged TM, $\langle \mathbf{T} \rangle$, is given by

$$\langle \mathbf{T} \rangle = \frac{1}{9}\sum_{t=1}^{9} \mathbf{T}_t \cdot \exp\left(-i\theta_t\right) \tag{14}$$

To provide a baseline, we tested the fluctuation level of 100 random input fields as the fibre was moved through the 9 configurations. To create each random input field, we randomized the phase of a 20×20 Cartesian grid of uniform intensity spots incident on the proximal facet MMF. The measured average fluctuation level was $F_l = 0.82 \pm 0.01$, as shown in Fig. 6. We compare this to the fluctuation levels measured from the most stable fluctuation eigenchannel calculated from the time-averaged TM, which yields $F_l = 0.19$, as shown in the main paper Fig. 4(e).

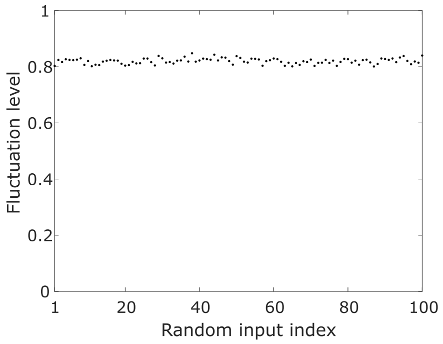

Figure 6. The measured fluctuation level of 100 random input fields

We might expect that the lowest temporal fluctuation eigenchannels of a flexible MMF may be similar to the eigenmodes of an ideal straight section of MMF. Indeed, we see from main paper Fig. 4(e) that the most stable temporal fluctuation eigenchannels are reminiscent of distorted versions of the MMF propagation invariant modes (PIMs) – and in particular PIMS that have low intensity near the middle of the core [10]. The low order PIMs, with intensity close to the axis of the fibre, have a more closely spaced set of phase velocities, and so more easily couple to one another when the fibre is perturbed. Therefore, it follows that the most stable fluctuation eigenchannels are not likely to include these low order PIMs – and hence they have low intensity on axis. A key advantage of our approach is that it returns the most stable channels without the need to project measured TMs into the PIM basis – as it is very challenging to experimentally identify the PIMs of a real MMF, due to the presence of aberrations in the surrounding optical system. Even with knowledge of the PIMs it is not obvious how to predict which fields will be most stable for a given set of fibres states – a problem that our method provides a way of tackling.

§8: **Numerical simulations of experiments and noise considerations**

Here we describe supporting numerical simulations of the methods demonstrated in the experiments in main paper Figs 1-3, where the artificial dynamic scattering medium is well controlled, and discuss the similarities and differences between our experimental results and the findings of our simulations.

SLM flicker: It is first worth detailing a key source of additional noise in our experiments: the effect of SLM flicker. The origin of liquid crystal SLM flicker is due to electric field pulse modulation (which also speeds up the response of the SLM). However, this effect results in an oscillatory global intensity modulation of light reflected from an SLM (even when the desired phase hologram is unchanging), and has the potential to artificially increase the measured fluctuation level in our experiments. Furthermore, DMDs can also exhibit a flicker effect when the mirrors switch between holograms. The effects of phase flicker varied in our different experiments:

Our unguided optimisation experiments optimise the input field directly using measured fluctuation levels, and so minimising spurious intensity fluctuations was crucial in this case. This experiment features two liquid crystal SLMs. We found the phase flicker exhibited by the SLM used to shape the input field (Hamamatsu) was negligible and so could be ignored. The unwanted effects of phase flicker were more pronounced from the SLM used to mimic the dynamic scattering medium (Holoeye), especially as light reflected from this SLM thrice. We were able to substantially suppress phase flicker in this experiment by synchronising the camera with this SLM, so that the camera was triggered to always capture images at the same point in the phase flicker cycle. Indeed, we found that if this synchronisation was turned off, spurious intensity fluctuations dominated our fluctuation measurements, and we were no longer able to optimise the input field to thread around the moving regions of the medium.

Our physical adjoint optimisation experiment features three SLMs (two liquid crystal Holoeye SLMs, and a DMD), which all exhibit flicker. We were unable to synchronise camera measurements to all three SLMs simultaneously. However, we found that our adjoint optimisation method worked without the need for camera synchronisation: since this approach relies on averaging optical fields, it is thus not strongly susceptible to global intensity fluctuations in the transmitted fields caused by SLM flicker. Nonetheless, when we monitor the fluctuations in the optimised fields, then there is an increased noise floor because of the three flickering SLMs. We suspect this contributes to the higher level of measured temporal fluctuations in this experiment compared to the other approaches, and also compared to our simulations (see below). Yet as shown in main paper Fig. 2(c), looking inside the scattering system shows very low power on dynamic parts of the sample.

Our time-averaged TM experiment features two liquid crystal SLMs (both Holoeye). The method is once again reliant on field averaging, and so is not strongly susceptible to global intensity fluctuations in the transmitted fields caused by SLM phase flicker. We experimentally observe lower levels of temporal fluctuations of the optimised fields in comparison with the physical adjoint

optimisation approach. We speculate this is due to the lower number of independently flickering SLMs in our time-averaged TM experiment (i.e. two SLMs in our time-averaged TM experiment versus three SLMs in our adjoint optimisation experiment).

Simulations of MPLC-based experiments: We now describe noiseless simulations of our three optimisation methods. We conduct scalar diffraction simulations, in which each phase plane is defined as an infinitely thin perfectly transmissive surface that imparts pixellated spatially varying phase delays to light transmitted through it. Diffraction of light through free-space between the planes is simulated using the angular spectrum method.

Unguided optimisation: Figure 7(a) shows a simulation of the unguided optimisation method demonstrated in Fig. 1 of the main paper. Since this simulation requires thousands of light field propagation calls, then to minimize computation time, we aim to keep the simulation area as small as possible while still reflecting experimental parameters. In this simulation the 3 planes are spaced by 1 cm – a slightly shorter distance than in experiments, chosen to prevent light 'falling off' the edge of the planes which leads to unphysical effects due to the periodic boundary conditions of the fast Fourier transform used in the angular spectrum method. We don't expect this to cause any qualitative different in the results. Each plane is 512×512 pixels, with a pixel pitch of 20μm. The lateral correlation length of the static random background phase pattern is 800 μm, and the red squares (of width and height 200 μm) highlight the dynamic region on each plane. Within this region, the lateral correlation length of the dynamic phase patterns is 80 μm, and a series of 20 random phase patterns is cycled. We shape the wavefront of the input field by optimising the phase of 4096 super-pixels to minimise the observed intensity fluctuations at the output. Input field optimisation is achieved using the unguided optimisation process described in the Methods.

Initialising the input field with random phases, we observe a convergence curve that plateaus at a non-zero fluctuation level (in this case at $F_l \sim 0.1$) – as also observed experimentally. Nonetheless we observe that very low intensity levels are found within the dynamic regions on each plane. Our simulation does not feature any noise sources, indicating that the algorithm has found a local minimum solution that substantially suppresses temporal fluctuations but has not found an optimal solution. We speculate this may be due to having control of only the phase of the field leaving the SLM. The structure of the optimal field propagating through the planes is highly speckled (i.e., features high spatial frequencies), which is similar in structure to that observed in our experiments.

Physical adjoint optimisation: Figure 7(b) shows a simulation of the physical adjoint optimisation method demonstrated in Fig. 2 of the main paper. In this case the lateral correlation lengths of the background and moving regions are the same as in Fig. 7(a), and the distance between the planes is 2 cm. In each iteration we average the transmitted field probing 20 randomly generated phase patterns placed within the 3 patches, and optimise the intensity and phase of 4096 super-pixels using the physical adjoint optimisation algorithm described in the Methods.

As in our experiments, we observe the intensity fluctuation level to plateau after ∼30 iterations. However, in these noiseless simulations the fluctuations reach a substantially lower level ($F_l \sim 0.02$) than in our experiments ($F_l \sim 0.2$). We speculate that the higher level of fluctuations observed in our experiments are mainly due to SLM phase flicker as described above. In addition, to work well, these experiments rely on the field measurement and field generation modules of the digital phase conjugation systems being in pixel-perfect alignment. Furthermore, the beams propagating forward and backward through the system must also be precisely co-aligned. Therefore, this experiment was also the most challenging from an alignment perspective – and small misalignments may also have contributed to the higher observed fluctuation levels in this experiment. Finally, the optimised field inside the sample takes a qualitatively similar form in these simulations to that found in our experiments – varying more smoothly than the fields found using unguided optimisation.

We also simulate the performance of the physical adjoint optimisation method in a more challenging scenario. Figure 10 shows a comparison of (a) the case with a single fluctuating patch per plane, to (b) a more challenging case when the area of fluctuating regions is increased to cover approximately half of the area of each plane. In this second case, we expect there to be no perfectly stable channels through the medium (as indicated by Fig. 12). While in (a) the fluctuation level converges to $F_l \sim 0.02$, in (b) we see that the fluctuation level converges to $F_l \sim 0.3$. Despite this, we see the field is still mainly confined to the static regions of each plane.

Time-averaged TM: Figure 7(c) shows a simulation of the physical adjoint optimisation method demonstrated in Fig. 2 of the main paper. Here the lateral correlation lengths of the background and moving regions are the same as in Fig. 7(a-b), and the distance between the planes is 2 cm. Each probe mode samples 20 randomly generated medium realisations (i.e., phase patterns placed within the 3 patches). The input basis is once again 4096 super-pixels.

We find $\sim 60\%$ of the fluctuation eigenchannels exhibit low fluctuation levels of $F_l \sim 0.03$, with the lowest fluctuation level ($F_l \sim 0.02$) matching that found using physical adjoint optimisation, and given by the highest time-averaged TM singular value. We find that the structure of the most stable field inside the sample takes a qualitatively similar form to those found using physical adjoint optimisation (i.e., smoothly varying), which also matches our experiments. The lower singular values show

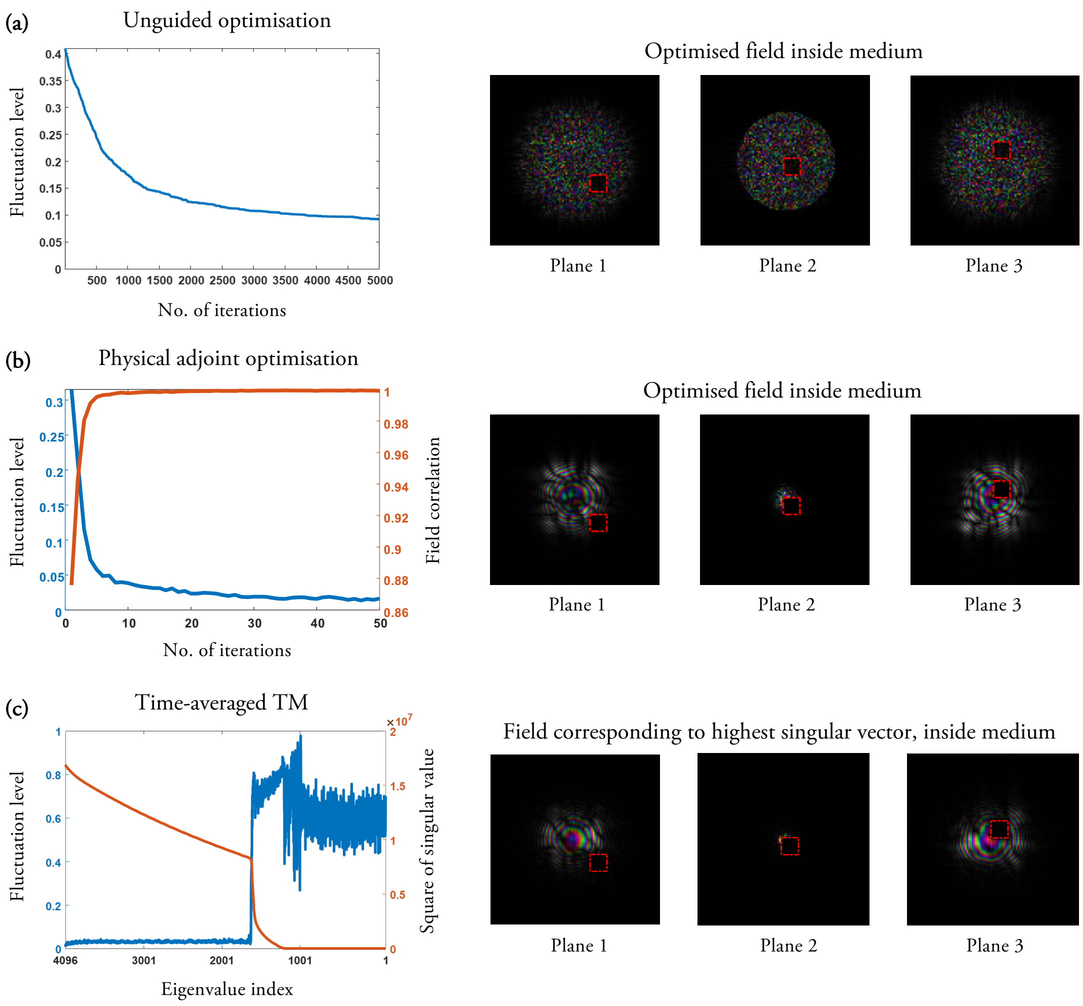

Figure 7. **Simulations of optimisation approaches**. (a) Unguided optimisation. (b) Physical adjoint optimisation. (c) Time-averaged TM.

much higher levels of temporal fluctuation.

§9: **Number of medium realisations per iteration required for physical adjoint optimisation**

In this section we study the performance of the physical adjoint optimisation approach as the number of medium realisations sampled per iteration is varied. We test the convergence rates of two variations of the physical adjoint optimisation procedure:

Figure 8 treats a model of a symmetrical algorithm in which the same iterative field update procedure is applied on both sides of the medium: on each side, the time-averaged field is phase conjugated and sent back through the medium. In this simulation the dynamic patches are $200\times200\,\mu$m in width. Lateral correlation lengths of the background and moving regions are both $800\,\mu$m – highlighting that our algorithm does not rely on the spatial frequency exhibited by the moving regions being higher than the spatial frequencies of the background medium. The distance between the planes is 2 cm, and we optimise the intensity and phase of 4096 super-pixels. Figure 8(a-e) shows convergence curves as the number of medium realisations probed in each direction per iteration is reduced from five to one. We see that convergence is achieved in the fewest iterations by averaging over five medium states per iteration. This is the case since a larger number of field averages provide a better estimate of how the field should be updated to reduce fluctuations on each iteration. We also see that convergence is achieved even averaging over only two medium realisations per iteration – albeit more noisily. This is because any degree of field averaging starts to push the algorithm in the right direction towards convergence. Finally, as shown in Fig. 8(e), performing no field averaging per iteration fails to converge as expected: we see the field correlation reduce and the fluctuation level increase in this case.

Figure 9 shows the convergence as the number of medium realisations probed in each direction per iteration is once again reduced from five to one, here treating a model of an asymmetrical algorithm in which on the right-hand-side of the medium the field average is phase conjugated and sent back, while on the left-hand-side of the medium, the field average is used as an update to the field sent in on the last iteration (as shown in main paper Fig. 2). All simulation parameters are the same as used

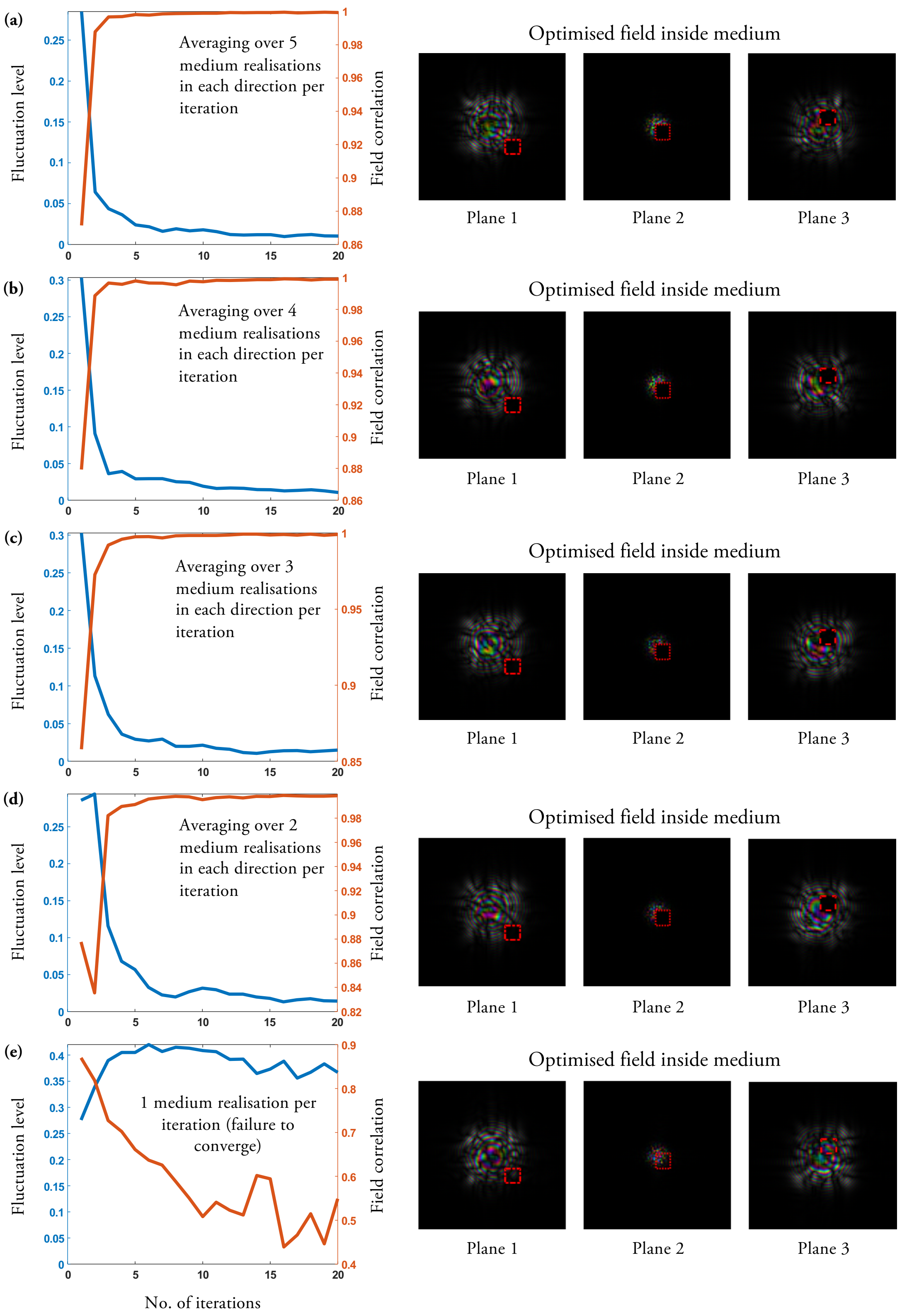

Figure 8. Performance of physical adjoint optimisation (symmetrical algorithm) as the number of medium realisations sampled per iteration is reduced.

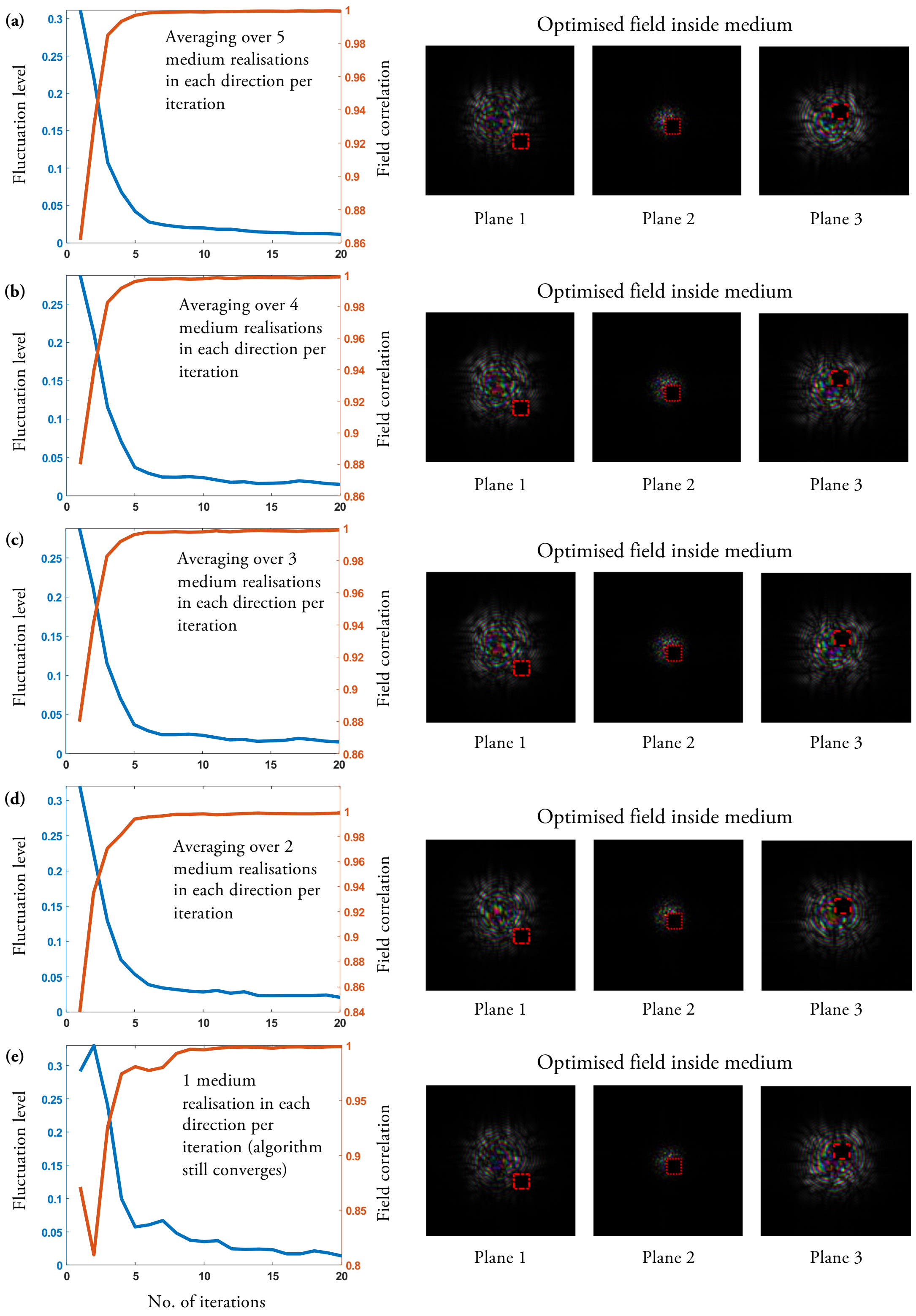

Figure 9. Performance of physical adjoint optimisation (asymmetrical algorithm) as the number of medium realisations sampled per iteration is reduced.

in Fig. 8. Interestingly, this means that a field averaging effect is accumulated over multiple iterations (since fields are added to those of previous iterations on the left-hand-side of the medium), so this algorithm still converges even without dedicated averaging on each iteration, as shown in Figure 9(e). It is worth highlighting that averaging over more medium realisations increases the time required per iteration, and so to minimise the overall convergence time, the number of medium realisations sampled per iteration may be optimised depending upon the particular scenario.

§10: **Physical adjoint optimisation with high levels of movement**

We now explore how physical adjoint optimisation performs when the level of motion in the forward scattering medium is increased. Firstly, in Fig. 10, we show that this algorithm can converge even when there are no perfectly stable channels within the medium. In this case we model three planes with 50% of each plane containing dynamically moving regions (illustrated by the yellow regions on the insets). Here there are no stable channels, as can be inferred from Fig. 12(c). Therefore the converged intensity fluctuation level is non-zero – however we see that the optimised field passes mainly through the static regions of each plane. In this simulation we optimise the intensity and phase of 4096 super-pixels, the planes are separated by 2 cm, the minimum feature size of the moving regions in 200 μm across, and the lateral correlation length of the background static region is 800 μm and the moving regions is 80 μm.

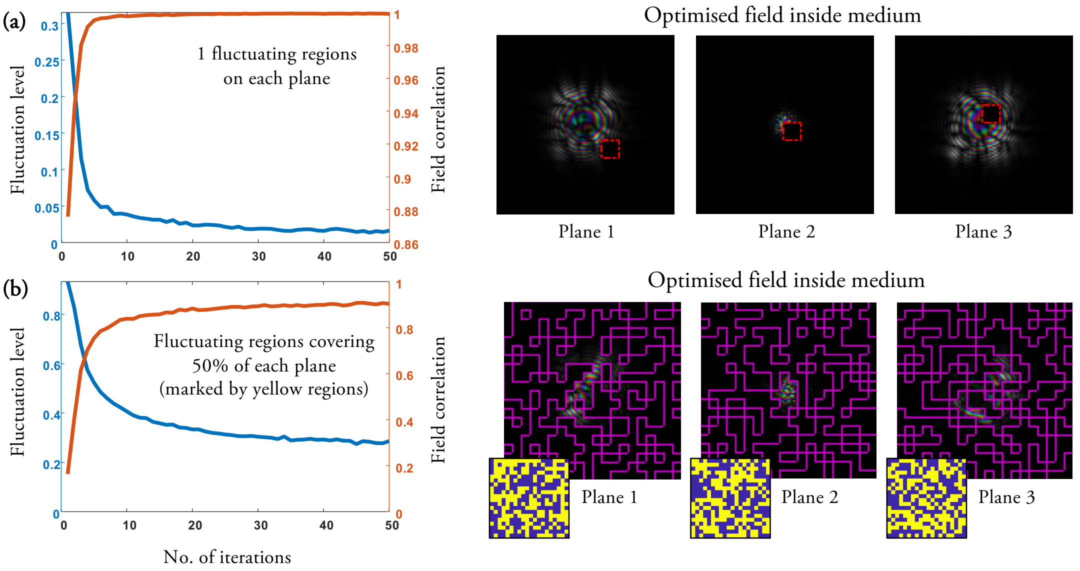

Figure 10. **Physical adjoint optimisation with increased area of fluctuating regions** (a) three fluctuating patches. (b) Approximately 50% of each plane is fluctuating. Boundary between fluctuating and static regions is highlighted by the pink line. Yellow highlights the fluctuating areas within the insets. In this case there are no perfectly stable channels through the medium.

Finally, we test how physical adjoint optimisation approach performs if there are no static regions, but the medium possesses regions of different decorrelation time. As before we model three planes: here the outer two planes are static, while the middle plane has a rapidly moving region within a 200×200 μm patch, surrounded by a background region with a spatial decorrelation length of 800 μm, that drifts sideways at different rates. In Fig. 11(a), the background drifts at a rate of 40 μm per iteration, corresponding to a fraction of 0.05 of the background decorrelation length per iteration. In this case the algorithm succsessfully guides light around the rapidly moving part of the middle plane. Figures 11(b-d) show the performance of the algorithm as the background rate is increased to 0.25 background decorrelation lengths per iteration (b), 0.55 decorrelation lengths per iteration (c) and 1 decorrelation length per iteration (d). The algorithm manages to converge to a solution avoiding the rapidly fluctuating patch in (b) and (c), albeit more noisily. However, the algorithm fails in (d), when the decorrelation rate of both the patch and the background region outpaces the iterative algorithm. This demonstrates that our physical adjoint optimisation approach can be applied when the entire medium is moving.

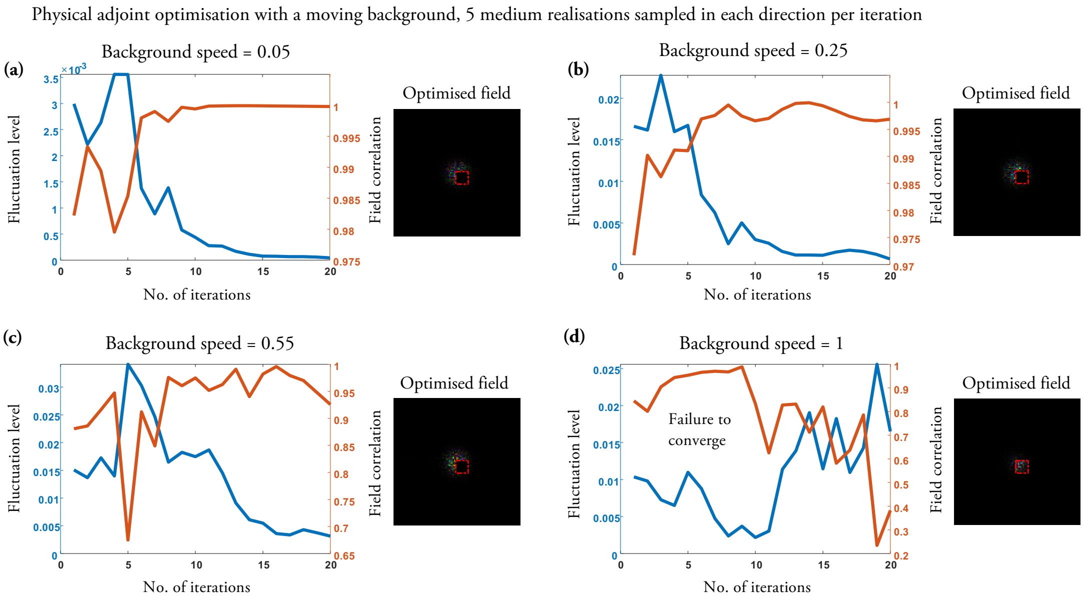

Figure 11. **Physical adjoint optimisation with a moving background: identification of slower moving channels**. In (a-c) light is successfully guided through the slower moving background region. In (d) the background region is moving too fast and so the algorithm fails.

§11: **Number of stable channels through lossless dynamic media**

In this section we investigate how the number of stable channels within a dynamic medium scales with the proportion of the medium that is in motion and its thickness. There is a wide range of possible geometries that scattering media can take, and so we construct a simple layered forward scattering model to study a generic case.

We simulate a dynamic scattering medium consisting of M perfectly transmissive phase planes, each of dimension $C = P \times P$ pixels. Here we set $P = 20$, thus we model a medium supporting $C = 400$ channels in total. We note that we have not observed any substantial differences in the results if P is increased to $P = 30$ ($C = 900$ channels) or decreased to $P = 10$ ($C = 100$ channels). Each phase plane is defined with a randomly chosen fraction of dynamic pixels, the phase of which can randomly fluctuate over time (with phase values on each timestep that are uncorrelated with the phase values at all other times), embedded within a static randomly chosen phase pattern. The phase delay imparted by each pixel is uncorrelated with its neighbours (and also has no longer range correlations). Each plane is connected to the next by a time-independent random scattering medium, represented by random unitary complex matrix. These matrices are fully connected, ensuring that light is fully spatially mixed when propagating from one plane to the next. This removes any dependency on the particular distribution of fluctuating pixels on each plane (for example, it makes no statistical difference if the fluctuating pixels are clustered together on each plane or randomly spread). This construction means we model a lossless medium that scatters all light forwards to the output plane. The effect of introducing loss is explored later (see below).

In our model, the time-dependent TM $\mathbf{T}(t)$ of the medium, at time t, represented in the canonical (pixel) input and output bases, is given by

$$\mathbf{T}(t) = \mathbf{H}_M \cdot \mathbf{P}_M(t) \cdot \mathbf{H}_{M-1} \cdot \mathbf{P}_{M-1}(t) \cdots \mathbf{H}_2 \cdot \mathbf{P}_2(t) \cdot \mathbf{H}_1 \cdot \mathbf{P}_1(t) = \overset{\curvearrowleft}{\prod_{m=1}^{M}} \left[\mathbf{H}_m \cdot \mathbf{P}_m(t)\right], \tag{15}$$

where $\mathbf{P}_m(t)$ is a diagonal matrix holding the phase delay imparted by each pixel of the mth phase plane at time t along its diagonal: if the cth pixel imparts a phase delay of θ_c, then the cth element of the diagonal of $\mathbf{P}_m$ (i.e., $P_{c,c}$) is given by $\exp(i\theta_c)$. Matrix $\mathbf{H}_m$ is the time-independent random complex unitary random matrix connecting planes m and $m+1$. The curved arrow above the product indicates that the matrix multiplications are written out labelling $m = 1$ on the right most matrices and with label m ascending to the left hand matrices (as written in the middle part of Eqn. 15).

We wish to calculate the time-averaged TM of this medium, $\langle \mathbf{T} \rangle_t$, and explore the distribution of stable channels as a function of the area fraction of dynamic pixels per layer, and the number of layers. To do this we can take a shortcut – exploiting the connection between the time-averaged TM of a dynamic medium, and the TM of an inhomogeneously absorbing medium [4, 5]. If the range of phase fluctuations of each dynamic pixel is 2π, we expect light that has scattered through these pixels to completely average away at the output (given enough medium realisations). Therefore, rather than simulating the TM as a function of t and

taking an average, we can replace the matrix elements corresponding to fluctuating pixels throughout the medium with zeros (i.e. making these regions fully absorbing). The TM of the resulting matrix product now directly calculates the time-averaged TM $\langle \mathbf{T} \rangle_t$, with the assumption that averaging has been carried out for long enough for all fluctuating fields to completely average away – in effect modelling the result of an infinite number of averages. To assess the number of stable channels, we calculate the singular value decomposition of $\langle \mathbf{T} \rangle_t$ (equivalent to calculating the eigendecomposition of matrix $\langle \mathbf{T} \rangle_t^\dagger \langle \mathbf{T} \rangle_t$ described in the main text). Singular values that are equal to 1 are associated with a stable channel through the dynamic medium.

Figure 12(a) shows how the singular value distribution of $\langle \mathbf{T} \rangle_t$ depends upon the number of layers (ranging from $M = 1$ to $M = 30$) for a medium with a dynamic region area fraction of 5% per layer. Each row of the heatmap depicts the singular value distribution for a medium consisting of a particular number of layers. As would be expected, we see that for a medium with a single layer, 95% of the channels (380/400) have a singular value equal to 1 and so are stable. As the number of layers increase, the fraction of stable channels reduces, as would also be expected. The reduction in the fraction of stable channels initially falls linearly with respect to the number of layers, but beyond $M \sim 15$ layers it shows a sub-linear decrease. Stable channels can extend deeply into this medium. For example, we find that $\sim 10\%$ of the modes remain highly stable to a depth of 20 layers. There are no perfectly stable channels remaining at a depth of 30 layers.

Figure 12(b) shows an equivalent heatmap for a higher dynamic region area fraction of 10% per layer. As would be expected, here we see the number of stable channels decreases more sharply with number of layers. In this case we find that $\sim 10\%$ of the modes remain highly stable to a depth of 10 layers, and there are no perfectly stable channels beyond a depth of 15 layers. Figure 12(c) shows how the fraction of stable channels decreases with number of layers as the dynamic fraction per layer ranges from 5% to 70%. Here we define a stable channel as one in which the 95% of the transmitted power avoids the dynamic regions of the medium (i.e., with a singular value of higher than a threshold of $0.95^{1/2}$). For example, when the dynamic area fraction per layer is 20%, we find that $\sim 9\%$ of the modes remain highly stable to a depth of 5 layers, and 1.5% are stable to a depth of 6 layers. Once the dynamic area fraction per layer is increased to 45%, we find that after 2 layers, the fraction of stable modes has fallen to $\sim 15\%$, with no stable channels to a depth of 3 layers.

In summary, our numerical study shows that stable temporal fluctuation eigenchannels can penetrate deeply into partially dynamic media, provided that the fraction of moving regions is relatively low.

§12: **Number of medium realisations needed to find stable channels using the time-averaged TM**

We next use our model to study how many medium realisations we must probe to construct a time-averaged TM capable of accurately identifying the most stable temporal fluctuation eigenchannels. We model a lossless 3-plane medium with two different levels of motion. We first simulate a scattering medium with a dynamic area fraction of 20% per plane. Figures 13(a-e) shows the effect of increasing the number of output field averages when calculating the time-averaged TM of this medium. We test $N = 1, 2, 5, 10$, and 30 medium realisations. Each plot features three lines: (i) The square of the singular values of the infinitely time-averaged TM (black line). This shows the relative level of coherently time-averaged intensity transmitted to the output plane for each of the true fluctuation eigenchannels, and as such is the same line in Figs. 13(a-e). In this case $\sim 40\%$ of the channels through the medium are stable, and thus the singular values associated with these channels have a relative intensity of 1. The stable channels are indicated by light blue shaded region. (ii) We also plot the square of the singular values of the time-

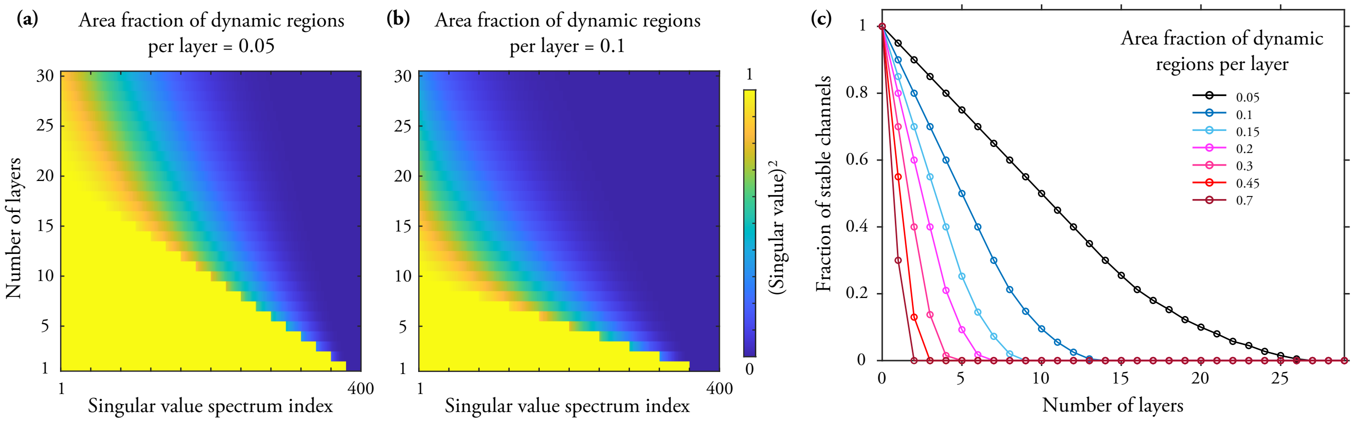

Figure 12. **Number of stable channels as a function of medium depth**. (a) Singular value distribution of $\langle \mathbf{T} \rangle_t$ as a function of the number of layers (ranging from $M = 1$ to $M = 30$) for a medium with a dynamic region area fraction of 5% per layer. (b) Singular value distribution of $\langle \mathbf{T} \rangle_t$ as a function of the number of layers (ranging from $M = 1$ to $M = 30$) for a medium with a dynamic region area fraction of 10% per layer. (c) The fraction of stable channels as a function of the number of layers, for a dynamic fraction per layer ranging from 5% to 70%.

averaged TM (red line), and (iii) the coherently time-averaged intensity transmitted through the medium by the singular vectors of the time-averaged TM (blue line). This blue line indicates the performance of the channels found using the time-averaged TM: where the blue line lies on top of the black line, the time-averaged TM reveals the true temporal fluctuation eigenchannels of the medium.

In Fig. 13(a), the singular values of the time-averaged TM when $N = 1$ (i.e., only a single realisation of the medium) are all equal to one, since the medium is lossless and fully forward scattering in our model. This one-medium-realisation time-averaged TM reveals no information about the true temporal fluctuation eigenchannels of the medium and so the singular vectors of the time-averaged TM are uncorrelated with the true temporal fluctuation eigenchannels of the medium (blue line is uncorrelated with black line).

Figure 13(b) shows the result of constructing the time-averaged TM by averaging the field scattered through just $N = 2$ realisations of the dynamic medium. We now see that the time-averaged TM correctly identifies all of the perfectly stable channels (blue line lies on top of black line within light blue shaded region). The reason for this is because after just two averages, the components of all output fields that have interacted with dynamic regions will have been reduced, while components of output fields that have not interacted with dynamic regions will not have been reduced. This demonstrates that if we are only interested in finding perfectly stable channels, and the medium is forward scattering and lossless, then as few as two averages will suffice (although we note that our simulation also does not include the effects of noise). However, when sampling only two medium realisations the tail of partially stable channels are not well captured. If these are of interest, then in this case increasing the number of medium realisations sampled to $N = 5$ substantially improves our ability to accurately identify these partially stable channels, as shown in Fig. 13(c) – where the blue line becomes more closely correlated with the black line. Figures 13(d-e) shows that this correlation progressively improves as the number of medium realisations sampled is further increased, tending to near perfect agreement by $N = 30$ averages.

We now explore the case where there are no perfectly stable fluctuation eigenchannels. We increase the dynamic area fraction to 50% on each of the three planes. The most stable true temporal fluctuation eigenchannel has a squared singular value equal to ~ 0.85, meaning that $\sim$15% of the transmitted energy interacts with dynamic parts of the sample. Our task here is to correctly identify the most stable channels. The result of increasing the number of medium realisations when measuring the time-averaged TM is shown in Figs. 13(f-j). Once again, a single measurement reveals no information about the temporal

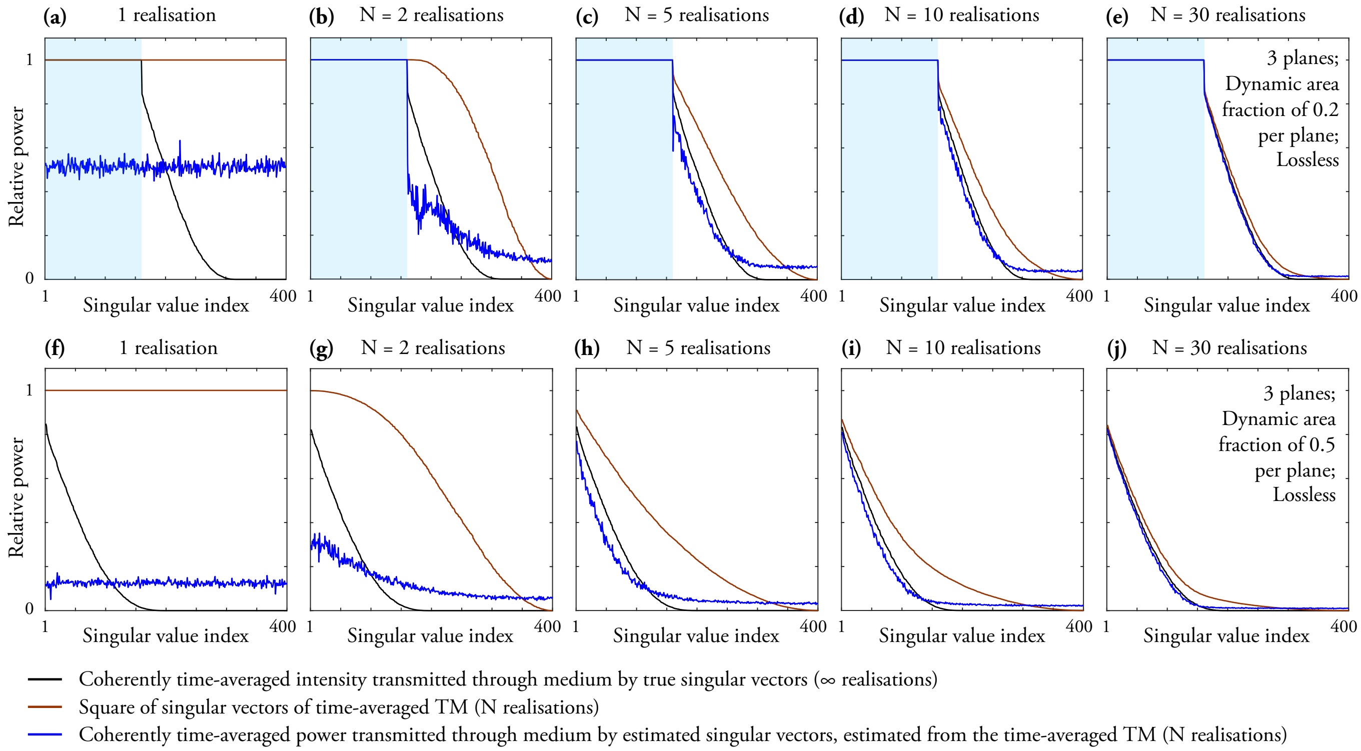

Figure 13. **Number of medium realisations used to calculate the time-averaged TM**. The upper row (a-e) shows how well the singular values can be estimated for a number of medium realisations ranging from N = 1 (i.e., no averaging) to N = 5, for a 3-plane medium with fluctuating regions covering 20% of each plane. The lower row (f-j) shows how well the singular values can be estimated for a number of medium realisations ranging from N = 1 to N = 5, for a 3-plane medium with fluctuating regions covering 50% of each plane.

fluctuation eigenchannels (Fig. 13(f)). Similar to the accurate identification of the tail of temporal fluctuation eigenchannels above, in this case more than $N = 2$ medium realisation must be sampled: sampling $N = 5 - 10$ medium realisations begins to enable accurate channel identification (i.e., the blue line approaches the black line). Using 30 medium realisations, the most stable channels are identified with good fidelity.

The effect of a limited phase fluctuation range: In Fig. 14 we investigate the effect of a reduction on the range of phase fluctuations undergone by the dynamic parts of the medium. We once again simulate a 3-plane medium with a dynamic area fraction of 20% on each plane – thus $\sim$40% of the temporal fluctuation eigenchannels are perfectly stable (light blue shading). Figures 14(a-c) show the performance of the time-averaged TM, calculated by sampling $N = 30$ medium realisations per input probe mode, as the phase fluctuation range is reduced from 2π (a) through π (b) to $\pi/5$ (c). We find that all of the stable channels can be identified in each case – this is because the even if the range of phase fluctuations is reduced, coherently averaging output fields always reduces the field components that have interacted with the dynamic parts of the medium, while those components that have avoided the dynamic regions are not reduced. However, reducing the level of phase fluctuation impacts our ability to correctly identify the tail of partially stable channels. In particular, it is no longer possible to accurately identify the least stable temporal fluctuation eigenchannels. This is because when the phase of the dynamically scattered light is not completely randomized, these components may not coherently average to zero, irrespective of the number of medium realisations sampled.

The effect of loss or incomplete scattering matrix measurement: Finally, we study the impact of loss within the medium, or equivalently, the inability to measure all of the scattered light. In our model we introduce loss into each plane by randomly setting a fraction of the diagonal matrix elements to zero. We keep track of which elements are designated as those with loss, and which are designated as fluctuating regions. We once again simulate a 3-layer medium with a dynamic area fraction of 20% and first test the effect of adding a loss fraction of 10% on each plane. In this case $\sim$ 15% of the temporal fluctuation eigenchannels are perfectly stable and navigate around both the fluctuating and lossy regions of the sample. We investigate to what extent it is possible to accurately identify these channels as we vary the number of medium realisations and the phase fluctuation range.

Figure 15(a) shows the performance of the time-averaged TM calculated by sampling $N = 2$ medium realisations per input mode, with a phase fluctuation range of 2π. We are able to accurately find the stable channels, but as with the lossless case above, with few medium realisations, the tail of partially stable channels is less well captured. In Fig. 15(b) we find all channels with good accuracy by increasing the number of medium realisations sampled per input mode to $N = 30$. In Figs. 15(c,d) we repeat the same tests with a reduced phase fluctuation range of $\pi/5$. Here we can once again correctly identify the stable channels using only $N = 2$ medium realisations sampled per input mode, however are unable to identify the tail of partially stable channels irrespective of the number of medium realisations probed per input mode.

Figures 15(e-h) show the same set of tests, but with the loss fraction increased to 40% per plane. In this case there are no perfectly stable channels. When the phase fluctuation range is 2π, it is possible to accurately identify the partially stable

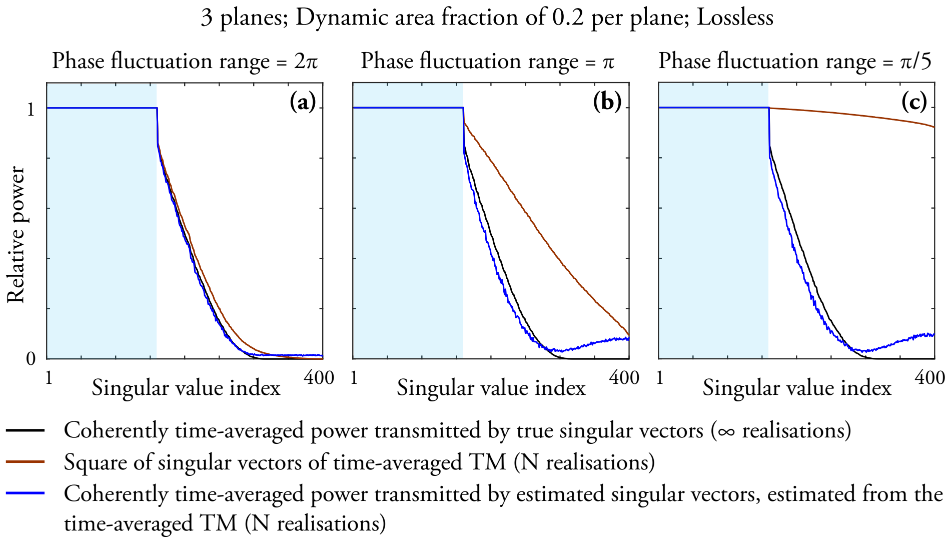

Figure 14. **Effect of phase fluctuation range for a lossless medium**. In each case $N = 30$ medium realisations are sampled, and the stable eigenchannels can be identified regardless of phase fluctuation level.

channels can be identified as long as enough medium realisations are sampled. However, when the phase fluctuation range is reduced to $\pi/5$, it is not possible to accurately identify the temporal fluctuation eigenchannels regardless of the number of medium realisations sampled. This is because the field fluctuations do not completely average away and so channels that avoid the lossy regions are preferentially identified over channels that avoid dynamic regions. A new approach is needed in this scenario, which is discussed below.

§13: **Exploration of different scattering regimes**

In the main text we gave a method for finding input fields that avoid dynamic regions of a scattering medium. This was based on an iterative procedure that increases a figure of merit equal to the overlap between all the output fields at different times

$$\mathcal{F}_1 = \frac{1}{N^2}\sum_{t,t'}\int_{\text{Out}} \mathrm{d}x v_t^*(x) v_{t'}(x) = \int_{\text{Out}} \mathrm{d}x |\langle v\rangle|^2, \tag{16}$$

which, as indicated can be equivalently understood as the square of the time averaged field, integrated over the output plane. To obtain an input field that avoids dynamic regions, the field simply needs to be successively averaged on input and output planes while the dynamic scattering region evolves in time. As shown above, each iteration of this forwards–backwards procedure is equivalent to a successive multiplication of the field by the absolute square of the time averaged transmission matrix $\langle \mathbf{T}\rangle^\dagger \cdot \langle \mathbf{T}\rangle$. As a result, repeatedly passing the field through the system converges to the singular vector of the average transmission matrix $\langle \mathbf{T}\rangle$ with the largest singular value.

Average transmission matrix: For systems with negligible back–scattering (as described in the main text), the norm of the forward propagating field is conserved between input and output planes so that the transmission matrix for the system at time t

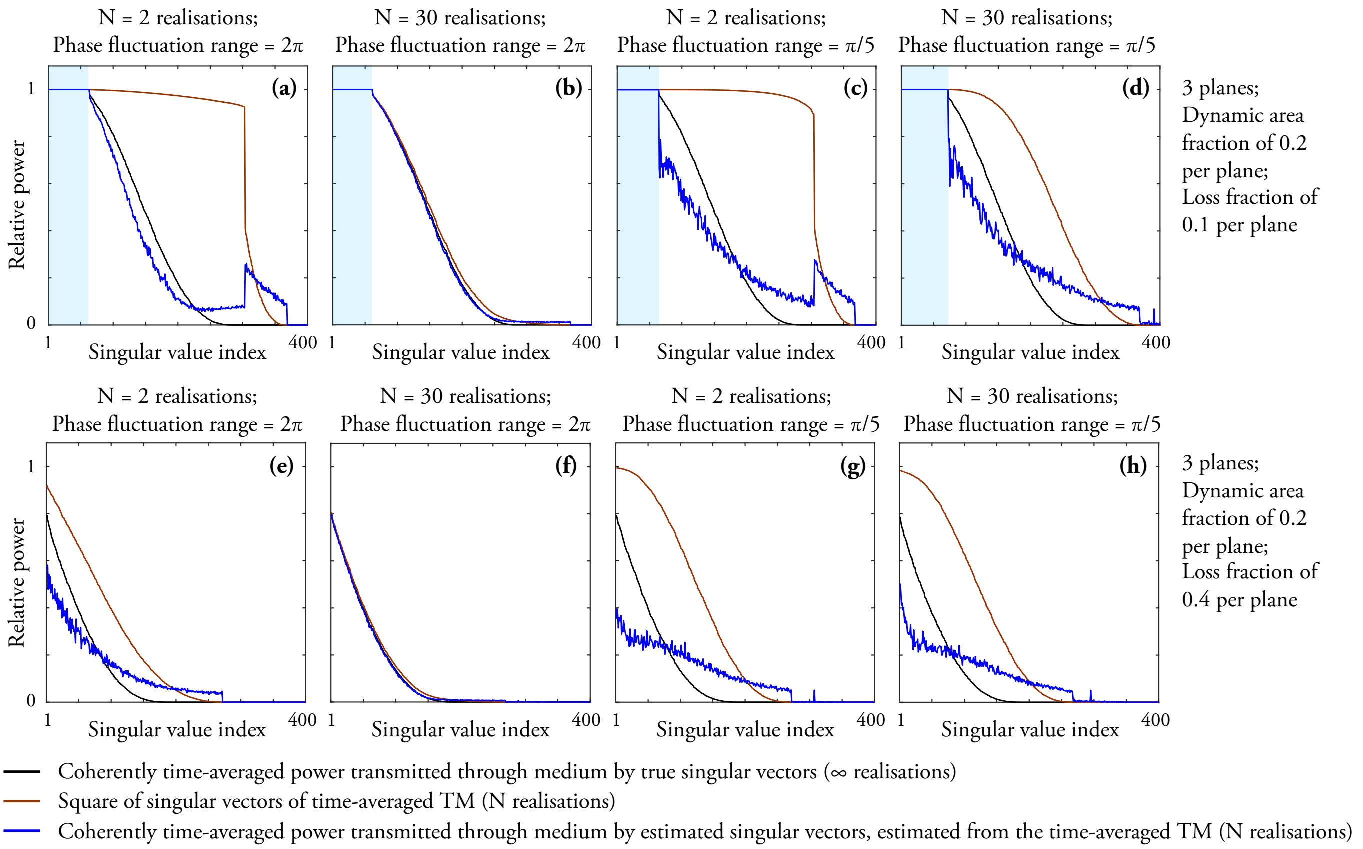

Figure 15. **Combined effect of phase fluctuation range and loss**. Upper row (a-e) shows a lossy scenario in which $\sim$15% of the channels thread around both fluctuating and lossy parts of the medium (thus yielding singular values of unity). These stable channels can be identified irrespective of the number of medium realisations (which can be as low as $N = 2$) and the fluctuation range. Lower row (e-h) shows a scenario of increased loss such that no channels exist that are able to thread around all fluctuating and lossy regions. In this case, the most stable channels can be indentified from the singular value decomposition of the time-averaged TM only if the phase fluctuation range is 2π rad.

is a unitary matrix $\mathbf{U}_t$

$$\boldsymbol{v}_t = \boldsymbol{U}_t \cdot \boldsymbol{u}. \tag{17}$$

Averaging the output field over N temporal snapshots of the system leads to the relation

$$\langle \boldsymbol{v} \rangle = \frac{1}{N} \sum_t \boldsymbol{v}_t = \left(\frac{1}{N} \sum_t \boldsymbol{U}_t \right) \cdot \boldsymbol{u} = \langle \boldsymbol{U} \rangle \cdot \boldsymbol{u}, \tag{18}$$

showing that the time averaged output field is given by the average transmission matrix acting on the input field $\boldsymbol{u}$.

A unitary matrix has a degenerate spectrum of singular values, all equal to unity. The effect of averaging a unitary matrix is to decrease these singular values below unity, which is clear if we examine the Rayleigh quotient of $\langle \boldsymbol{U} \rangle^\dagger \cdot \langle \boldsymbol{U} \rangle$ with a normalized field $\boldsymbol{\nu}$

$$\begin{aligned} \frac{\boldsymbol{\nu}^\dagger \cdot \langle \boldsymbol{U} \rangle^\dagger \cdot \langle \boldsymbol{U} \rangle \cdot \boldsymbol{\nu}}{\boldsymbol{\nu}^\dagger \cdot \boldsymbol{\nu}} &\leq \sigma_{\max}^2 \\ &= \boldsymbol{\nu}^\dagger \cdot \left(\frac{1}{N^2} \sum_{t,t'} \boldsymbol{U}_{t'}^\dagger \cdot \boldsymbol{U}_t \right) \cdot \boldsymbol{\nu} \\ &= \frac{1}{N} + \frac{1}{N^2} \sum_{t \neq t'} \mathrm{Re}[\boldsymbol{\nu}^\dagger \cdot \left(\boldsymbol{U}_{t'}^\dagger \cdot \boldsymbol{U}_t \right) \cdot \boldsymbol{\nu}] \\ &\leq \frac{1}{N} + \frac{N(N-1)}{N^2} = 1 \end{aligned} \tag{19}$$

where $\sigma_{\max}$ is the largest singular value of the time averaged transmission matrix $\langle \boldsymbol{U} \rangle$ and equality with unity in the final line only occurs for the subspace where $\boldsymbol{U}_t \cdot \boldsymbol{\nu}$ is independent of t. As discussed above, maximizing the figure of merit $\mathcal{F}_1$ ensures the input vector equals the singular vector of $\boldsymbol{U}_t$ with the largest singular value. We can thus see that this makes the output field approach the same function for all values of t, thus reducing output field fluctuations. This optimized field will thus avoid the dynamic region of the scattering medium. Fig. 16 uses randomly generated unitary matrices to demonstrate how the reduced singular values indicated in Eq. (19) correspond to fields that are concentrated on the modulated scattering region.

Average scattering matrix: In systems where back–scattering is not negligible, the transmission matrix is not unitary. In this case we can maximize the average transmitted field (16) in at least two ways, either avoiding the dynamic region of the material (thus reducing output fluctuations), *or* by reducing back–scattering, increasing the overall norm of the field on the output plane. If we perform the successive averaging of the field in a system with significant back–scattering, then if the dynamically scattered field components do not average to zero on the detection plane, the final field may therefore not avoid the dynamic region and therefore still exhibit large output fluctuations in time.

However, provided material dissipation is negligible the *scattering* matrix is unitary. The time averaged scattering matrix can therefore be used to avoid dynamic regions, just as described for the transmission matrix in the previous section. The only complication is that all scattered light must now be collected. Confining the scattering medium between a left and right plane, the scattering matrix at time t can be written as

$$\boldsymbol{S}_t = \begin{pmatrix} \boldsymbol{T}_t & \boldsymbol{R}_t \\ \overline{\boldsymbol{R}}_t & \boldsymbol{T}_t \end{pmatrix} \tag{20}$$

where $\boldsymbol{T}_t$ is the transmission matrix, and $\boldsymbol{R}_t$ and $\overline{\boldsymbol{R}}_t$ are the reflection matrices for incidence from the left and right planes respectively. The input–output relation (17) takes the same form

$$\boldsymbol{v}_t = \boldsymbol{S}_t \cdot \boldsymbol{u}, \tag{21}$$

but the input vector $\boldsymbol{u}$ is now twice as long, including incident fields from both the left and right of the medium. Time averaging the output on both sides of the scattering medium, sending the conjugate of this average back through the medium and averaging for a second time, the average output equals

$$\langle \boldsymbol{v} \rangle = \langle \boldsymbol{S} \rangle^T \cdot \langle \boldsymbol{S} \rangle^* \cdot \boldsymbol{u}^*. \tag{22}$$

Repeating this process—just as for the transmission matrix—the output field approaches the singular vector of the scattering matrix with the largest singular value. As shown in Figs. 17 and 18, these singular vectors of the averaged scattering matrix can be used to construct input fields that either avoid, or are concentrated within the dynamic region of the material.

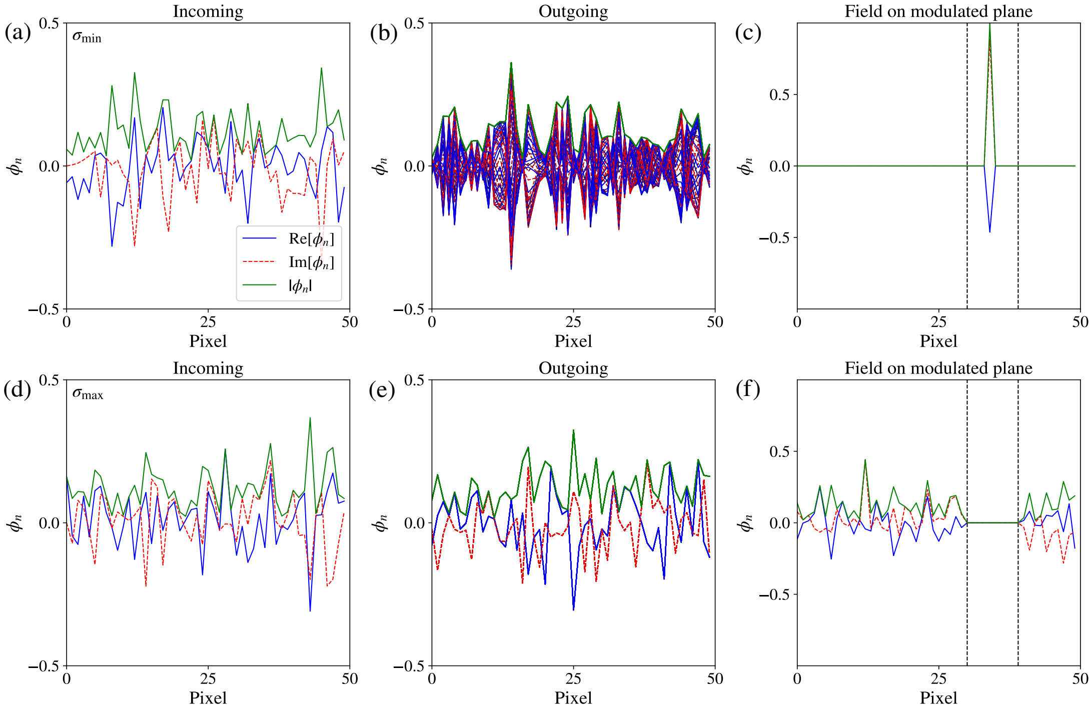

Figure 16. Averaging a unitary transmission matrix: We constructed a set of $N = 20$, 50×50 unitary matrices $\boldsymbol{U}_t$, each equal to a product of 10 randomly generated unitary matrices $\boldsymbol{u}_n$ ($\boldsymbol{u}_n = \exp(i\boldsymbol{H}_n)$, with $\boldsymbol{H}_n$ a random Hermitian matrix), $\boldsymbol{U}_t = \boldsymbol{u}_N \ldots \boldsymbol{D}_t \ldots \boldsymbol{u}_2 \cdot \boldsymbol{u}_1$, representing wave propagation through a material. Within this product we have inserted a diagonal matrix $\boldsymbol{D}_t$, which applies a phase shift to a chosen region of the field (within the black dashed lines in panels (c) and (f)). The averaged transmission matrix $\langle\boldsymbol{U}\rangle$ in Eq. (18) is calculated by averaging $\boldsymbol{U}_t$ for the 20 different random choices of phase $\boldsymbol{D}_t$. The singular vectors $\boldsymbol{u}$ of $\langle\boldsymbol{U}\rangle$ with minimum and maximum singular values are shown in panels (a) and (d), and the outputs $\boldsymbol{v}_t = \boldsymbol{U}_t \cdot \boldsymbol{u}$ for different values of t are superimposed in panels (b) and (e). The field with reduced fluctuation avoids the modulated region (panel (f)), while the large fluctuations (b) are due to focusing onto the modulated region (panel (c)).

Methods without averaging: We have shown that—even in cases where there is significant back–scattering—our repeated forwards–backwards propagation can be used to avoid dynamic regions of a material. However, in the general case we must collect all the scattered light, average over time, and send it back though the system. Yet in many situations we may only have the transmission matrix available. Here we address the question of how we can tailor the input field to avoid a dynamic region of a material using only the transmission matrix.

In this section we describe our dynamic material using the discrete dipole method [2], restricting ourselves to a scalar field in 2D, and randomly distributing N_p particles of maximum polarizability $\alpha = 4\mathrm{i}/k_0^2$ (see e.g. [1]) in a $25\lambda \times 25\lambda$ box, with an input source of extent $\Delta y = 75\lambda$ on the left of this box and a similar output plane on the right. Taking just the particles inside a circle of radius $R = 5\lambda$ within the box (the dynamic region), we generated $N = 55$ configurations of these particles, which represent different time snapshots of the dynamic medium.

Considering the time averaged transmission from the left plane to the right, both the fluctuations of the medium and the scattering away from the output plane make the transmission matrix non–unitary. As shown in Figs. 19 and 20, repeated propagation of the field between input and output planes, followed by time averaging at each plane does not fully eliminate the output fluctuations, and in the case of Fig. 19 the field does not avoid the dynamic region. This confirms the behaviour discussed above, due to the non–unitarity of the transmission matrix.

We now give an example of an alternative approach for avoiding dynamic regions based on a different figure of merit. Instead of maximizing the norm of the time–averaged field on the output plane, we instead more directly look to minimize the deviation of the field at every moment from its time average,

$$\mathcal{F}_2 = \sum_{t,t'} \int_{\mathrm{Out}} \mathrm{d}x |v_t(x) - v_{t'}(x)|^2. \tag{23}$$

Again we derive the procedure for modifying the input source through making a small change in the source field $u(x) \rightarrow$

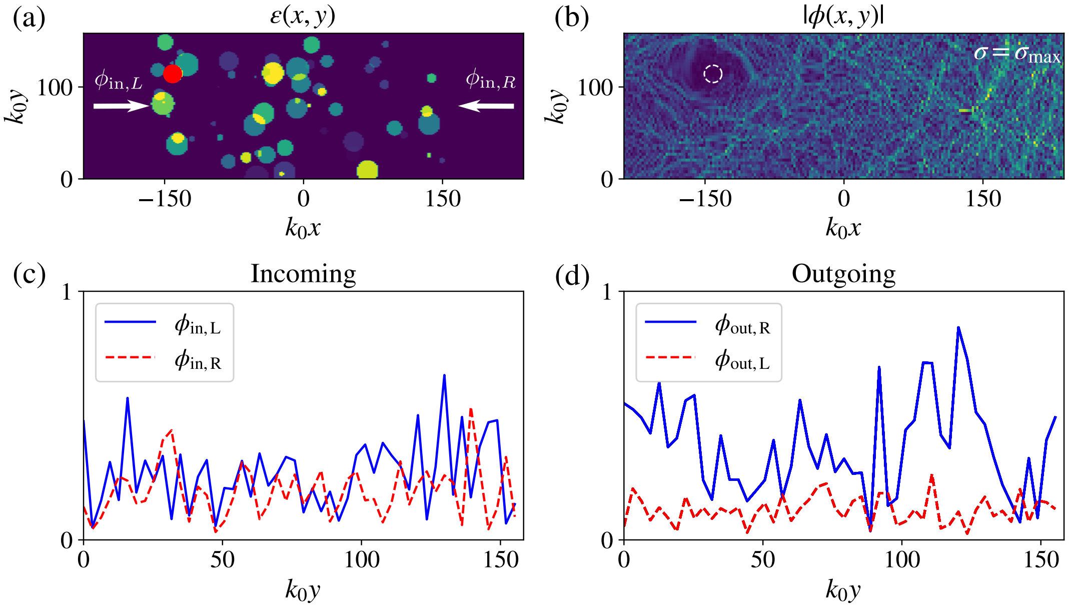

Figure 17. Time averaged scattering matrix for avoiding a dynamic region: (a) Permittivity profile constructed as a series of randomly placed discs of random radius $r \in [0.1\lambda, 2.1\lambda]$ (λ =wavelength) and $\Delta\varepsilon \in [0,1]$, one of which (red) is modulated randomly in time. (b) Assuming periodic boundary conditions along the y axis and discretizing the region $y \in [0, 25.1\lambda]$ we applied the transfer matrix method [3] to calculate both the average scattering matrix, and propagate the singular vector with maximum singular value ($|\phi_{\mathrm{in}}|$ in panel c) through the profile, clearly showing the field avoids the dynamic region of permittivity. (d) All output fields ($|\phi_{\mathrm{out}}|$) for all realizations of the profile shown in (a), illustrating negligible fluctuations.

$u(x) + \delta u(x)$. This leads to a small change in the output fields $v_t(x) \to v_t(x) + \delta v_t(x)$, which in turn modified (23) to,

$$\delta\mathcal{F}_2 = 4\,\mathrm{Re}\left[\sum_t \int_{\mathrm{Out}} \mathrm{d}x (v_t^*(x) - \langle v^*(x)\rangle)\delta v_t(x)\right]. \tag{24}$$

Using the expression for the change in the field due to a (in this case small) change in the source field, $\delta v_t(x) = \int_{\mathrm{In}} \mathrm{d}x' G_t(x,x')\delta u(x')$ and reciprocity $G_t(x,x') = G_t(x',x)$, the figure of merit (24) changes by

$$\delta\mathcal{F}_2 = 4\,\mathrm{Re}\left[\sum_t \int_{\mathrm{In}} \delta u_2(x') \int_{\mathrm{Out}} \mathrm{d}x\, G_t(x',x)(v_t^*(x) - \langle v^*(x)\rangle)\mathrm{d}x'\right]. \tag{25}$$

given that we want $\mathcal{F}_2$ to *decrease*, this implies the change in the source field should be chosen as

$$\delta u_2(x') = -|\delta u_2| \exp\left(-i \arg\left[\sum_t \int_{\mathrm{Out}} \mathrm{d}x\, G_t(x',x)(v_t^*(x) - \langle v^*(x)\rangle)\,\mathrm{d}x'\right]\right) \tag{26}$$

The modified figure of merit (23) leads to an iterative scheme (26) where we must record individual snapshots of the field on the output plane. The conjugate of the difference between the each snapshot and the average field, $v_t^* - \langle v\rangle$, must then be sent back through the dynamic medium, synchronizing each return field with the configuration of the medium at time t. This is only a feasible strategy if the dynamics of the medium is much slower than the time taken to return the field, or the dynamics of the medium is periodic, where the field can be recorded in one period and returned to the input plane in the next.

Figures 21 and 22 show the results of implementing (26) in our simulations based on the discrete dipole method, in two different scattering regimes. Evidently, in both cases the fluctuations $\mathcal{F}_2$ are reduced by around 3 orders of magnitude and are clearly much less than the corresponding output fields in Figs. 19 and 20. The only effect of increasing the number of scatterers is an overall reduction in intensity of the field on the output plane, due to increased back–scattering and scattering out of the top and bottom of the simulation area.

Note that there are many other choices of figures of merit that reduce output fluctuations in the field. However, in general they lead to optimization strategies similar to that described in this section: ones where the field must be passed between input and

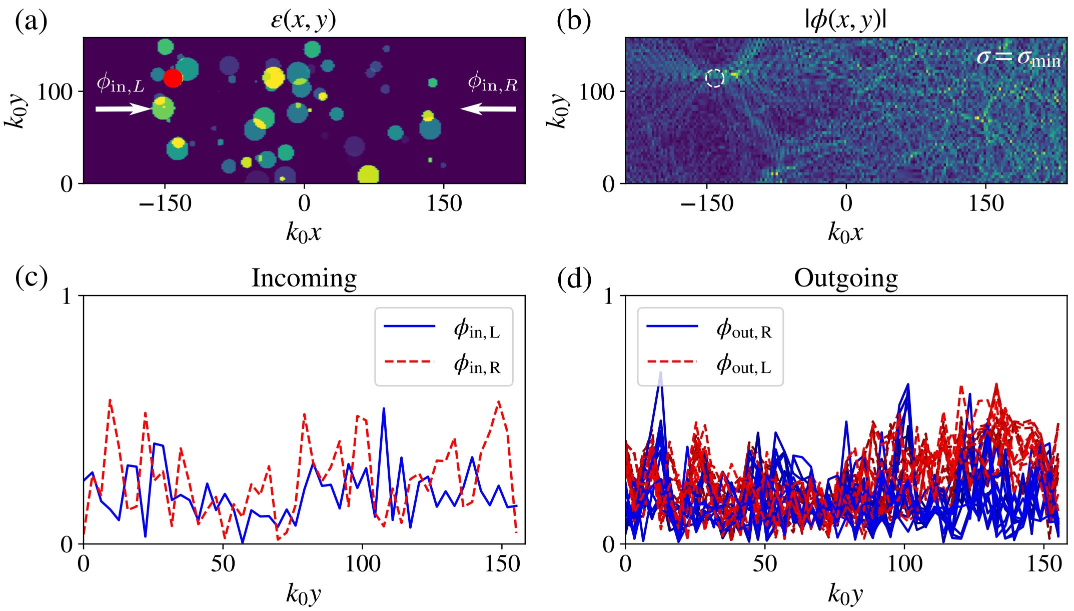

Figure 18. As in Fig. 17, but for the minimum singular value of the time averaged scattering matrix. Panel (b) shows the concentration of the field onto the dynamic region, producing the significant output fluctuations shown in panel (d).

output planes in synchronicity with the motion of the medium. Despite the restrictions of experimentally implementing these algorithms, in highly scattering systems where there is only access to a transmission measurement, they can lead to a much improved performance compared to the figure of merit (3) used in the main text.

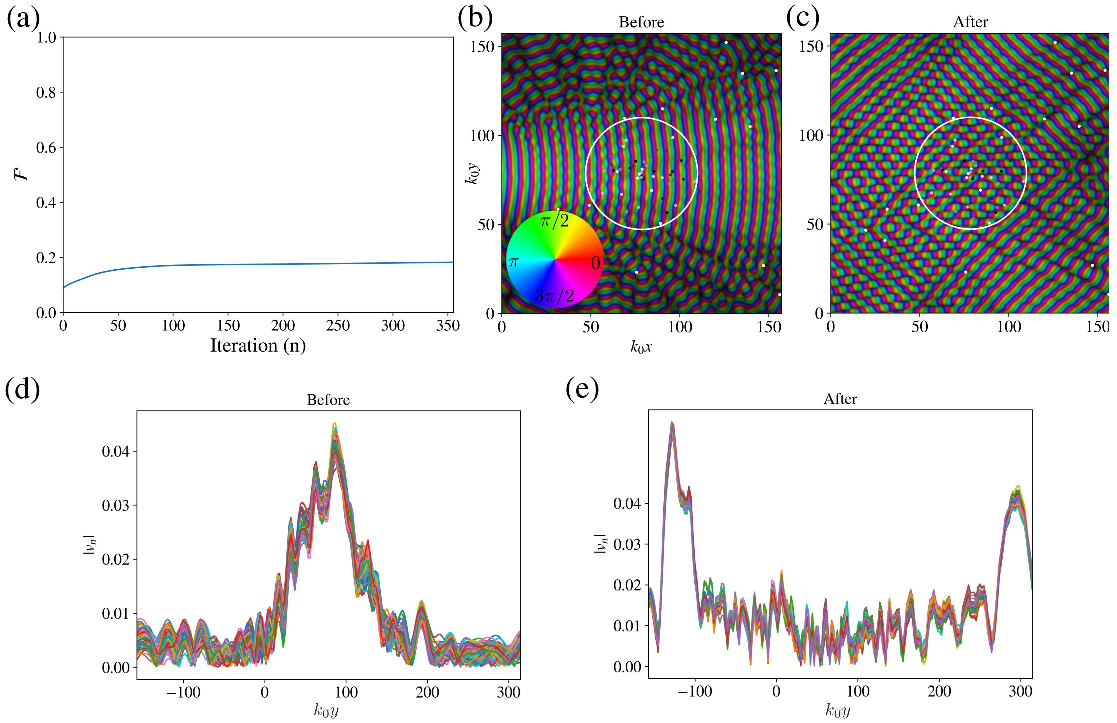

Figure 19. Optimization of an input field to avoid a dynamic region of a material. We randomly distribute 15 particles in a $25\lambda \times 25\lambda$ area (white dots in panels (b) and (c)). Those falling within the 5λ radius circle shown have their position within the circle randomized 55 times (shaded with a different shade of grey for each realization), which represent the different time snapshots. For an initial input field in the form of a Gaussian with variance $\sigma^2 = 50/k_0$ the output fields for all different time are superimposed in panel (d). Following the optimization procedure described in §3, the figure of merit $\mathcal{F}_1$ is increased as shown in panel (a) (note figure of merit $\mathcal{F}_1$ does not include integration measure, hence the large values), resulting in the optimzied fields shown in panels (d) and (e).

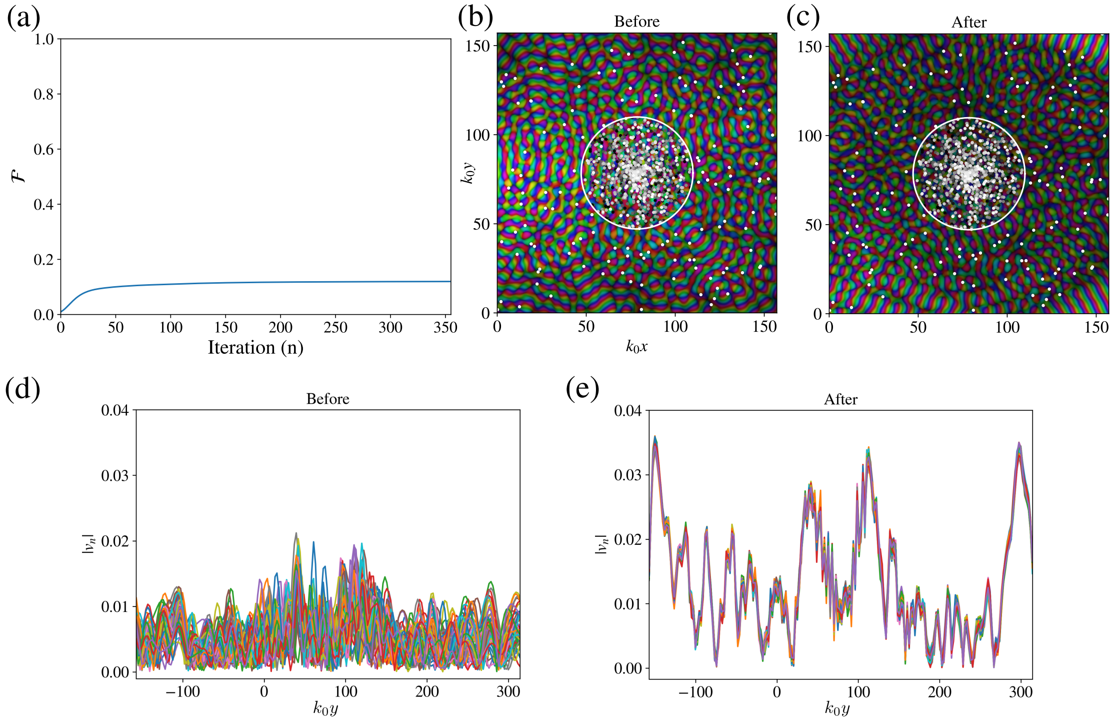

Figure 20. For the same parameters as Fig. 19, but for $N_p = 150$ particles.

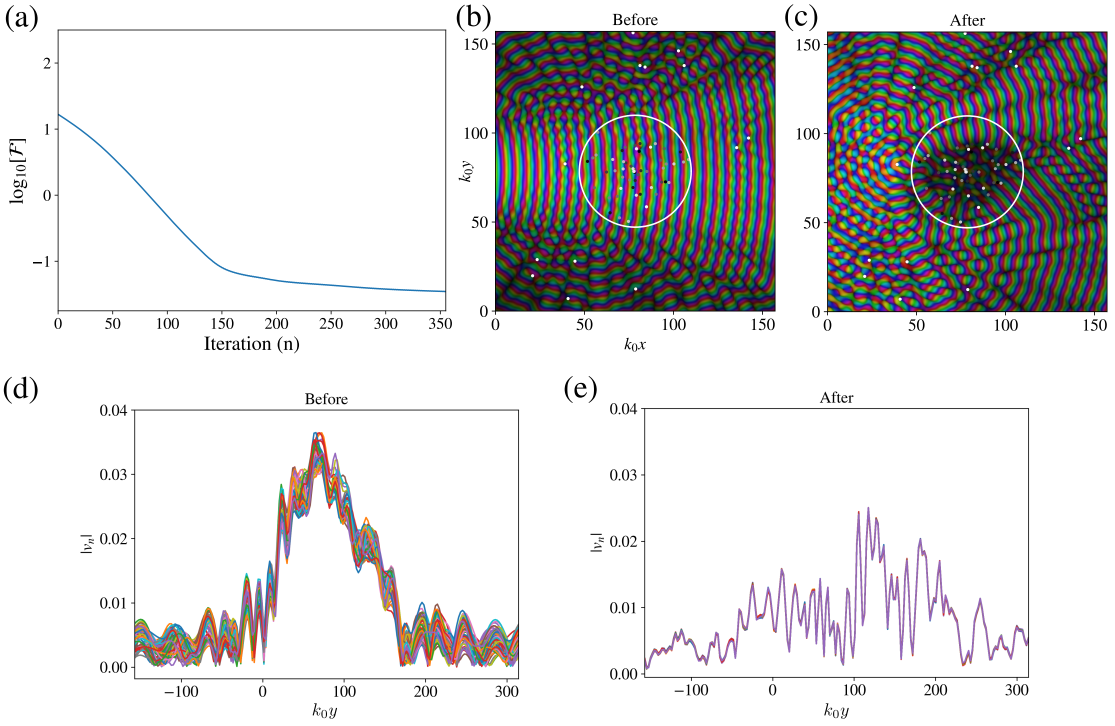

Figure 21. Parameters as in Fig. 19, for $N_p = 15$ and using the figure of merit given by Eq. (23) and the source updated according to Eq. (26). Note that here the optimization *reduces* the figure of merit, and panel (a) is accordingly plotted on a logarithmic scale.

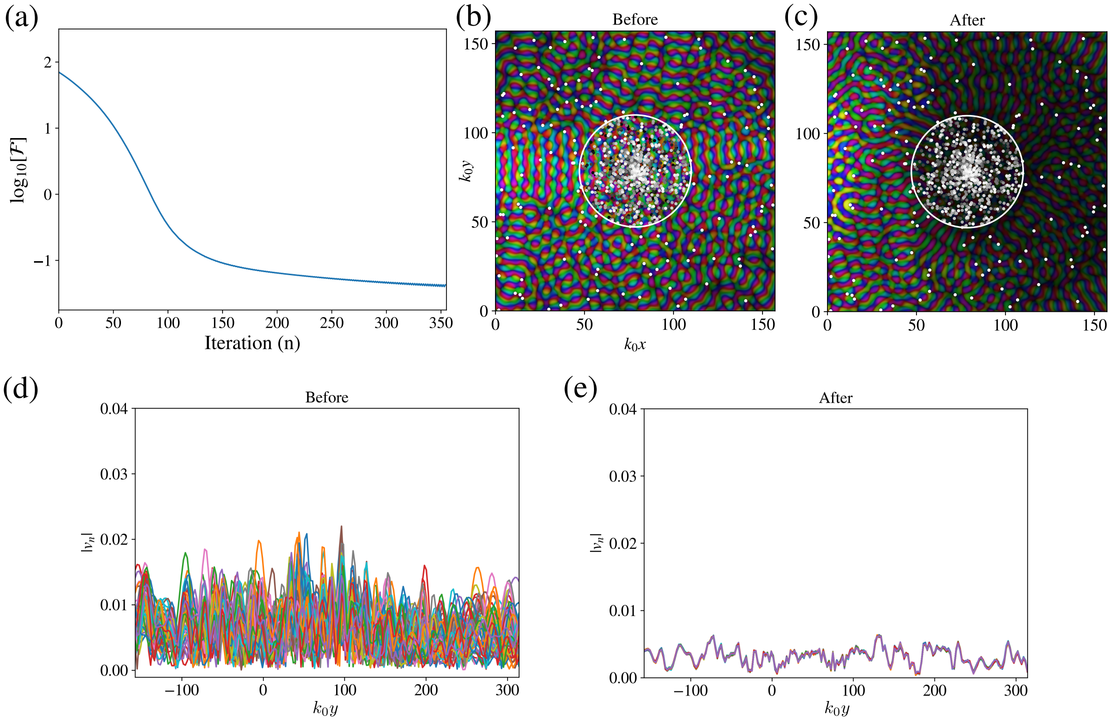

Figure 22. Parameters as in Fig. 21, but for $N_p = 150$ particles.

§14: **Description of supplementary movies**

Movie 1: Camera frames showing the time-dependent intensity profile of light transmitted through a dynamic scattering medium. Left panel: initial field. Right panel: Optimised field, found using unguided optimisation. We observe that the optimised field exhibits much lower levels of time-dependent fluctuations than the initial field. Scale bar shows relative intensity.

Movie 2: Camera frames showing the time-dependent intensity profile of light transmitted through a dynamic scattering medium. Left panel: initial field. Right panel: Optimised field, found using physical adjoint optimisation. We observe that the optimised field exhibits much lower levels of time-dependent fluctuations than the initial field. Scale bar shows relative intensity.

Movie 3: Camera frames showing the time-dependent intensity profile of light transmitted through a dynamic scattering medium. Each panel shows the transmitted light when a particular fluctuation eigenchannel is excited (the eigenchannel index is labelled above each panel). We observe that the light transmitted through the high index eigenchannels fluctuates the least, while light transmitted through low index eigenchannels exhibits much higher levels of temporal fluctuations. Scale bar shows relative intensity.

Movie 4: Camera frames showing the time-dependent intensity profile of light transmitted through a dynamic scattering medium. Left panel: the result of attempting to form a focus at the output using the conventional inverse TM. Right panel: the focus generated through the same sample, formed by exciting only the top 100 most stable fluctuation eigenchannels. In both cases we scan the position of the focus over 4 different positions. We observe that the focus created using the stable eigenchannels is enhanced: it has a higher contrast, and exhibits lower levels of temporal fluctuations. Scale bar shows relative intensity.

Movie 5: Camera frames showing the time-dependent intensity fluctuations of the most stable fluctuation eigenchannels projected through the MMF as it is moved through its 9 configurations (top row). The bottom row shows the fluctuations of unstable eigenchannels (left two panels) and random inputs (right two panels).

[1] J. R. Capers, S. J. Boyes, A. P. Hibbins, and S. A. R. Horsley. *Designing the collective non-local responses of metasurfaces*, Communications Physics, **4**(1):209 (2021).

[2] B. T. Draine and P. J. Flatau. *Discrete-dipole approximation for scattering calculations*, *JOSA A*, **11**(4):1491–1499 (1994).

[3] J. Lekner. *Theory of Reflection of Electromagnetic and Particle Waves*, Springer, (1987).

[4] S. F. Liew, and H. Cao. *Modification of light transmission channels by inhomogeneous absorption in random media*, *Optics Express*, **23**(9):11043–11053 (2015).

[5] S. Raktim, A. Yamilov, and H. Cao. *Enhancing light transmission through a disordered waveguide with inhomogeneous scattering and loss*, *Applied Physics Letters*, **110**(2):11043–11053 (2017).

[6] J. A. Davis, D. M. Cottrell, J. Campos, M. J. Yzuel, and I. Moreno. *Encoding amplitude information onto phase-only filters*, *Applied Optics*, **38**(23):5004–5013 (1999).

[7] W. H. Lee. *Binary computer-generated holograms*, *Applied Optics*, **18**(21):3661-3669 (1979).

[8] C. K. Mididoddi, R. A. Lennon, S. Li, and D. B. Phillips. *High-fidelity off-axis digital optical phase conjugation with transmission matrix assisted calibration*, *Optics Express*, **28**(23):34692-34705 (2020).

[9] Li, S., Horsley, S.A.R., Tyc, T., T., Čižmár, T., and Phillips, D. B. *Memory effect assisted imaging through multimode optical fibres*, *Nature Communications* **12**(1):3751 (2021).

[10] Plöschner, M. and Tyc, T. and Čižmár, T. *Seeing through chaos in multimode fibres*, *Nature Photonics* **9**(8):529-535 (2015).

[11] Tomáš Čižmár, Michael Mazilu, and Kishan Dholakia. *In-situ wavefront correction and its application to micromanipulation*, *Nature Photonics*, **4**(6):388, (2010).
